# Recent Progress in Electronic Skin

**DOI:** 10.1002/advs.201500169

**Published:** 2015-07-14

**Authors:** Xiandi Wang, Lin Dong, Hanlu Zhang, Ruomeng Yu, Caofeng Pan, Zhong Lin Wang

**Affiliations:** ^1^ Beijing Institute of Nanoenergy and NanosystemsChinese Academy of SciencesBeijing100083P. R. China; ^2^ School of Materials Science and EngineeringGeorgia Institute of TechnologyAtlantaGA30332‐0245USA

**Keywords:** electronic skin, flexible, multifunctional device, pressure mapping, tactile sensor

## Abstract

The skin is the largest organ of the human body and can sense pressure, temperature, and other complex environmental stimuli or conditions. The mimicry of human skin's sensory ability via electronics is a topic of innovative research that could find broad applications in robotics, artificial intelligence, and human–machine interfaces, all of which promote the development of electronic skin (e‐skin). To imitate tactile sensing via e‐skins, flexible and stretchable pressure sensor arrays are constructed based on different transduction mechanisms and structural designs. These arrays can map pressure with high resolution and rapid response beyond that of human perception. Multi‐modal force sensing, temperature, and humidity detection, as well as self‐healing abilities are also exploited for multi‐functional e‐skins. Other recent progress in this field includes the integration with high‐density flexible circuits for signal processing, the combination with wireless technology for convenient sensing and energy/data transfer, and the development of self‐powered e‐skins. Future opportunities lie in the fabrication of highly intelligent e‐skins that can sense and respond to variations in the external environment. The rapidly increasing innovations in this area will be important to the scientific community and to the future of human life.

## 1 Introduction

**Figure 1:**
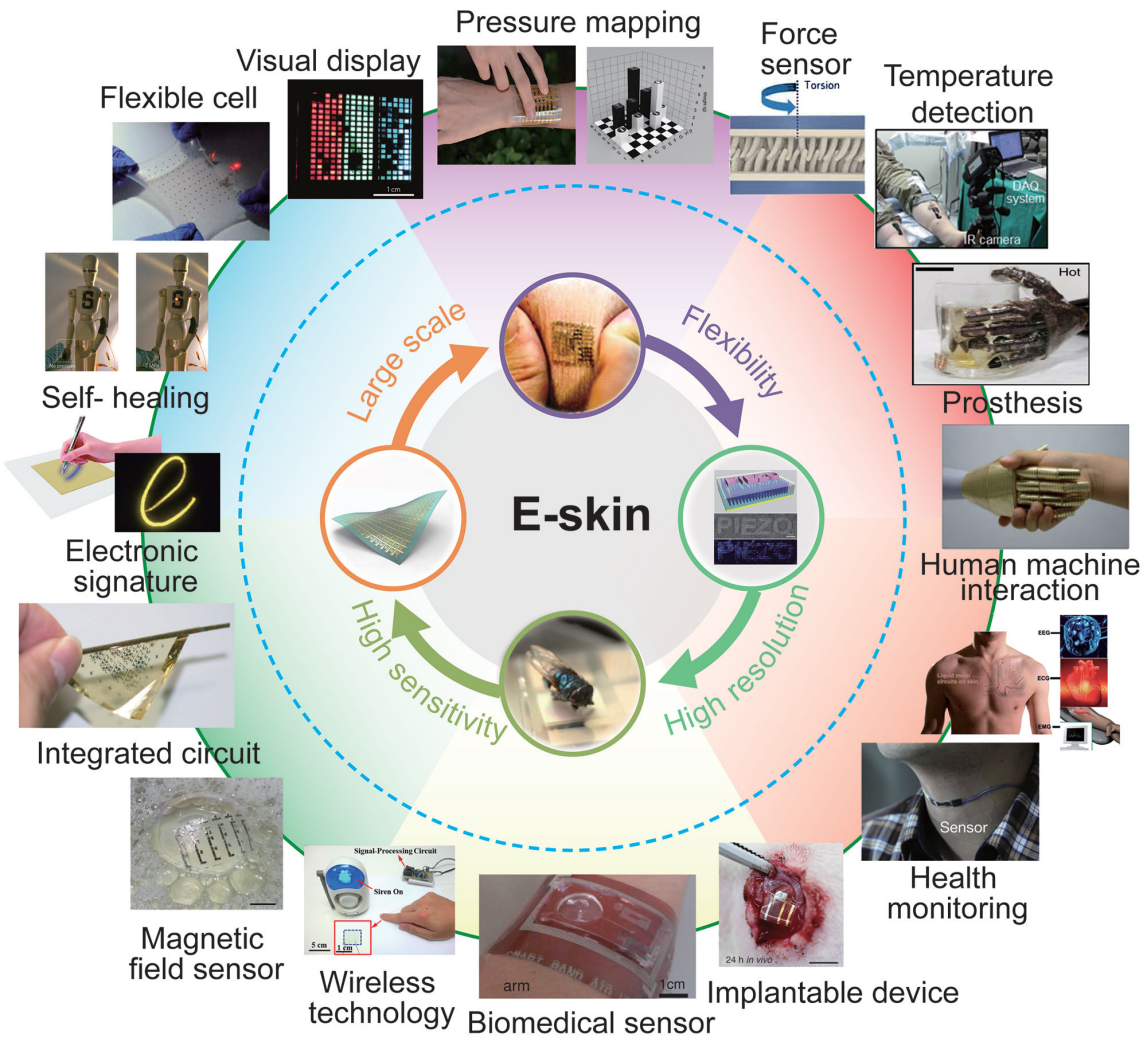
Characteristic properties and diverse functions or applications of recently developed devices for e‐skins. “Large scale:” Reproduced with permission.^12^ Copyright 2013, Macmillan Publishers Ltd. “High sensitivity:” Reproduced with permission.^31^ Copyright 2010, Macmillan Publishers Ltd. “High resolution:” Reproduced with permission.[[qv: 15a]] Copyright 2013, Macmillan Publishers Ltd. "Pressure mapping:” Reproduced with permission.[[qv: 13b]] Copyright 2014, Macmillan Publishers Ltd. Reproduced with permission.^30^ Copyright 2015, Macmillan Publishers Ltd. “Force sensor:” Reproduced with permission.^10^ Copyright 2012, Macmillan Publishers Ltd. “Prosthesis:” Reproduced with permission.[[qv: 9b]] Copyright 2014, Macmillan Publishers Ltd. “Magnetic field sensor:” Reproduced with permission.^106^ Copyright 2015, Macmillan Publishers Ltd. “Integrated circuit:” Reproduced with permission.^105^ Copyright 2008, American Association for the Advancement of Science. “Self‐healing:” Reproduced with permission.[[qv: 11a]] Copyright 2012, Macmillan Publishers Ltd. “Flexible cell:” Reproduced with permission.^75^ Copyright 2013, Macmillan Publishers Ltd. “Visual display:” Reproduced with permission.^61^ Copyright 2013, Macmillan Publishers Ltd. “Health monitoring:” Reproduced with permission.[[qv: 1c]] Copyright 2014, Royal Society of Chemistry. Reproduced with permission.^19^ Copyright 2014, Macmillan Publishers Ltd. “Temperature detection:” Reproduced with permission.^96^ “Biomedical sensor:” Reproduced with permission.^111^ “Implantable device:” Reproduced with permission.[[qv: 107a]] “Electronic signature:” Reproduced with permission.^74^ “Flexibility:” Reproduced with permission.[[qv: 9a]] Copyright 2011, American Association for the Advancement of Science. “Wireless technology:” Reproduced with permission.^82^ Copyright 2014, American Chemical Society. “Human machine interaction:” Reproduced with permission.[[qv: 1g]] Copyright 2012, Cambridge University Press.

Mimicry of the comprehensive properties of human sensing via electronic methods is a highly interesting topic for the development of artificial intelligence and human–machine interactive electronics.^1^ Significant achievements and mature technologies have been demonstrated for emulation systems, including the use of a high‐resolution camera to replace human sight and the substitution of a hi‐fi stereo component system for human voice. However, profound challenges remain in tactile‐sensing simulation of the human skin characteristics with high resolution, high sensitivity, and rapid response.^2^ In addition, electronic skin (e‐skin) should possess the ability to distinguish among diverse mechanical forces and to sense temperature or humidity simultaneously (similar to human skin) to maintain the physiological balance between the body and the ambient surroundings, all of which present significant challenges for the application in robotics and prosthetics (**Figure** **1**).^3^

As early as the 1970s, several researchers had explored the potential application of tactile‐sensing simulation and had demonstrated certain inspiring touch sensors, e.g., a prosthetic hand with tactile feedback and a personal computer with a touchscreen, but with low resolution and rigid materials.^4^ Up to the 1990s, significant breakthroughs were achieved in flexible and stretchable electronics for various applications using flexible materials.^5^ Especially over the last decade, tactile sensors with improved performance have been continuously developed based on different physical transduction mechanisms, including piezoresistivity, capacitance, and piezoelectricity.^6^ For example, large‐scale and flexible pressure‐sensitive sensor arrays based on transistors not only possess excellent mechanical and electrical properties but also enormously reduce the crosstalk between pixels for precise mapping of pressure distribution.^7^ Additionally, the use of oriented piezoelectric nanowires (NWs) and nanobelts (NBs) provides an opportunity to fabricate highly integrated tactile sensor arrays capable of high‐resolution tactile mapping beyond that of human sensing.^8^

Attempts to fabricate multifunctional e‐skins with human‐like perceptive characteristics have also received attention to satisfy the additional wide‐ranging industrial demands.^9^ These artificial intelligent e‐skins with diverse sensing modules can simultaneously differentiate among various physical stimuli from the complex external environment, including strain, twist, temperature, and humidity.^10^ The self‐healing and/or self‐powered devices are especially favorable for next‐generation multifunctional e‐skins.^11^ Recently, ultrathin, flexible, and multipurpose sensors with high‐density integrated circuits and other chemical or physical sensors have been developed for applications to robotics, health monitoring, and medical implant services.^12^

In this review, we primarily focus on the current strategies and technologies for the exploitation of e‐skins and the desired performance. First, we introduce the fundamental transduction mechanisms commonly used in e‐skins. Major technical improvements in e‐skins are described for the acquisition of excellent stretchability, sensitivity, and resolution properties for accurate tactile sensing. Additionally, we highlight the most recent breakthroughs in multifunctional e‐skins and summarize recent development trends and application forecasts.

## 2 Transduction Mechanisms

Recently, electronic skin that can sense pressure, strain, shear forces, and twist deformation has attracted attentions. Effective signal transduction that converts external stimuli into an analog electronic signal is an important component of accurate quantitative monitoring. Traditional transduction methods (e.g., piezoresistivity,^13^ capacitance,^14^ and piezoelectricity^15^ are widely used in different types of sensors, and other transduction methods (e.g., optics, wireless antennas, and triboelectricity) are undergoing rapid development to meet new challenges and opportunities that will broaden the applications of e‐skin to robotics, prosthesis, and human–machine interaction.^16^ The details of selected methods are presented in this section.

### 2.1 Piezoresistivity

Piezoresistive sensors enable transduction of force variations into changes in resistance that can be easily detected by an electrical measuring system; these sensors are widely used due to their simple device design and readout mechanism.^17^ The most common approaches to obtaining the resistance dependence of pressure‐sensitive sensors include changes in the contact resistance between conductive materials and changes in the conductive path in conductive elastic composites.

**Figure 2:**
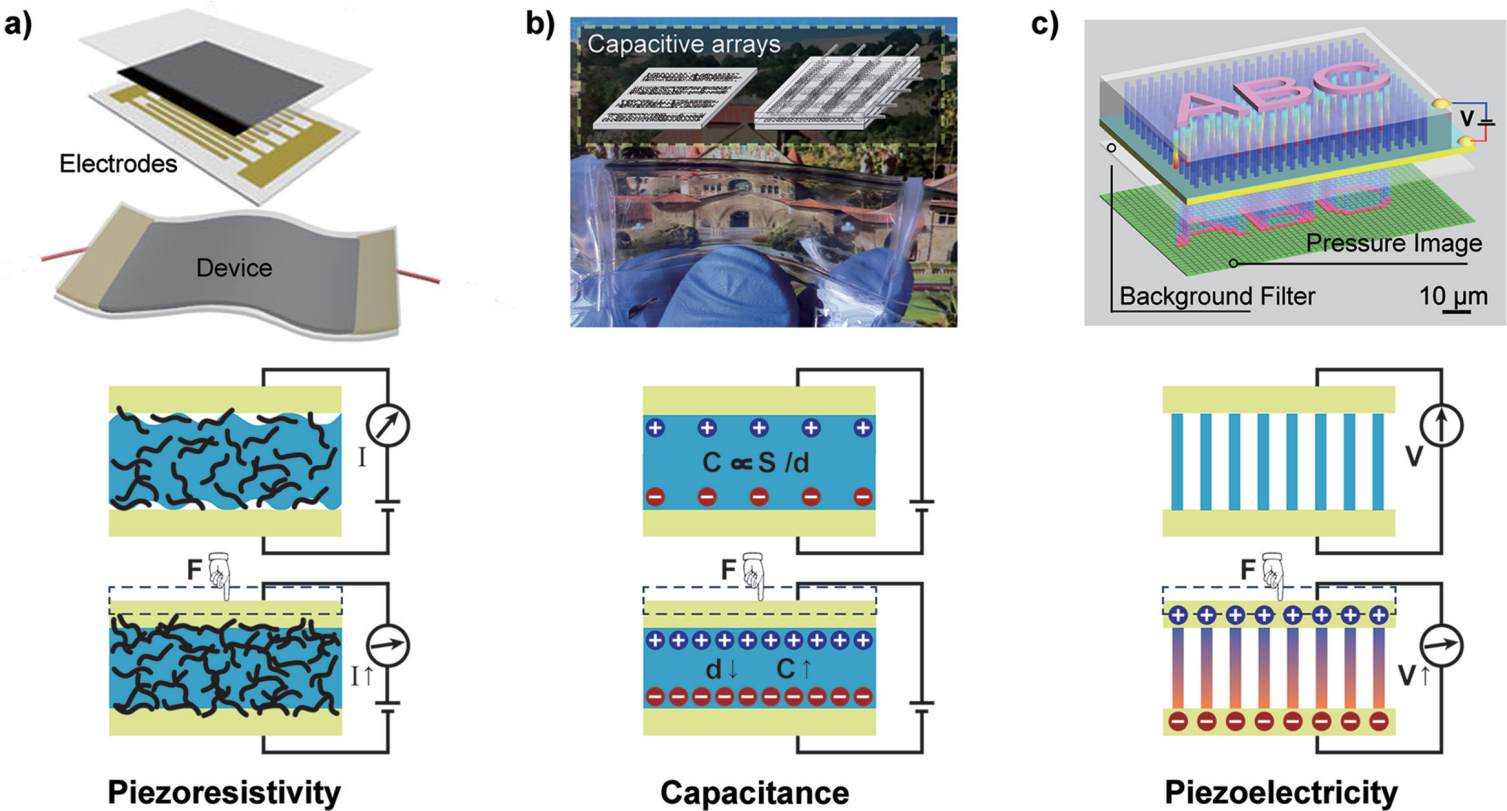
Schematic illustrations of three common transduction methods and representative devices: a) piezoresistivity, b) capacitance, and c) piezoelectricity. Reproduced with permission.[[qv: 15a]],^21^, ^29^ Copyright 2013, 2014, and 2011, respectively, Macmillan Publishers Ltd.

The change in the contact resistance caused by the variation of the contact area between two components is proportional to the square root of the force,^18^ which allows the sensors to detect lower pressures and expands the usable range. Certain sensors that mimic biological sensory organs based on the change in contact resistance were satisfactorily demonstrated for the detection of gas vibrations.^19^ A fast‐response and low‐creep strain sensor based on the structural deformation of gaps, islands, and bundles in aligned single‐wall carbon nanotubes (SWCNTs) under different strains permitted improvement of the strain response by up to 280%.^20^ Recently, Cheng and co‐workers developed a highly sensitive wearable sensor based on variable resistances between gold NWs and interdigitated electrode arrays on an elastic substrate, thus providing a wide working range from 13 to 50 000 Pa (**Figure** **2**a).^21^

Piezoresistive elastic composites consisting of conductive fillers embedded in soft polymer matrixes have been widely used because of their ease of preparation and low cost.^22^ Conventional pressure‐sensitive rubber (PSR) sheets with carbon black are always integrated with transistors to create a pressure‐sensitive active matrix for mapping the pressure distribution, but these sheets also encounter low sensitivity and large hysteresis.^23^ Currently, many types of filler materials and elastomer matrixes, such as metallic particles,^24^ graphene,^25^ and carbon nanotubes^26^ have been investigated to improve mechanical and electrical properties. Recently, Bao and co‐workers demonstrated an ultra‐sensitive pressure sensor array with rapid response and a low‐pressure regime, i.e., as low as 1 Pa, using an elastic hollow‐sphere microstructured conductive polymer.[[qv: 13b]]

### 2.2 Capacitance

The capacitance (*C*) of a parallel plate capacitor, the ability to store a charge, is described by *C = εA/d*, where *ε* is the dielectric constant, and *A* and *d* are the area and the distance between the two electrodes, respectively. Traditional capacitive sensors have been commonly used to measure different forces by monitoring the changes in *A* and *d* for which the applied pressure or shear force can easily result in the variation of the distance or the area between the two conductive plates.^27^ The major advantage of these sensors is the characteristic of high strain sensitivity for the detection of a static force with low‐power consumption and the precise modification of the device design by analysis of the simple governing equation.^28^ Bao and co‐workers demonstrated a transparent and stretchable capacitive sensor array based on carbon nanotube electrodes on elastic substrates that is sensitive to both pressure and strain (Figure 2b).^29^

Recently, capacitive sensors with variable effective dielectric constants have attracted significant interest for tactile sensing along with the rapid development of flexible field‐effect transistors.^30^ A typical transistor structure includes a gate electrode, source‐drain electrodes, a semiconductor, and a gate dielectric. Using the microstructured elastic dielectric layers in which the capacitance dramatically and rapidly changes after applied pressure because of the existence of air gaps, many transistors with high‐pressure sensitivity were demonstrated that also provided an obvious drain current signal output for the accurate detection of the pressure distribution.^31^

### 2.3 Piezoelectricity

Piezoelectricity refers to the production of electrical charges in certain materials under mechanical force due to the occurrence of electrical dipole moments. Dipole moments can be derived from the deformation of oriented noncentrosymmetric crystal structures^32^ or porous electrets with long‐lasting charges in the pores.[[qv: 15d]],^33^ This approach is widely used to convert mechanical stresses and vibrations into electrical signals via piezoelectric materials with high sensitivity, rapid response, and a high piezoelectric coefficient (*d*_33_).

Piezoelectric inorganics typically exhibit high *d*_33_ values but low flexibility, whereas piezoelectric polymers display the opposite. To exploit flexible piezoelectric pressure sensors with high *d*_33_, certain groups have attempted a variety of approaches, including the construction of thin films of piezoelectric inorganics on flexible substrates,^34^ the use of piezoelectric polymers or inorganics/polymer composites,^35^ and the construction of steady piezoelectrets.^36^ Recently, oriented piezoelectric NWs and NBs with intrinsically high piezoelectricity and good mechanical stability have attracted growing interest for the development of integrated high‐resolution sensing arrays for e‐skin. For example, Wang and co‐workers demonstrated flexible sensor arrays using oriented ZnO NWs that could map the pressure distribution with high sensitivity and high spatial resolution, comparable to those of the human skin's sense of touch (Figure 2c).[[qv: 15a]]

### 2.4 Other Transduction Mechanisms

In addition to the mentioned methods, other novel transduction methods have been investigated for the expansive application of e‐skin. Optical pressure sensors, which can cause modification of the light intensity or wavelength between the light source and the terminal detectors with applied pressure, have attracted attentions for application in touch screens and visual displays.^37^ In wireless transduction devices, the force‐induced resonant frequency of the resonant circuit is changed due to the variation of the effective coupling capacitance.[[qv: 16c]] These sensors are widely used in human–machine interactions and wireless health monitoring. Another interesting triboelectric sensor based on electrostatic induction and contact electrification enables the device to monitor the touch action without the requirement for an external power supply, which is promising for the creation of self‐powered sensors.[[qv: 11b]]

## 3 High‐Performance E‐Skin: Design and Fabrication

Good flexibility and stretchability are significant for skin to maintain its pressure‐sensing ability under complex mechanical deformation and have inspired the development of flexible and stretchable pressure sensors for e‐skins. High flexibility can be obtained simply by employing thinner flexible substrates, whereas stretchability requires techniques that are more sophisticated, such as an island‐bridge structure design. In addition, key research goals are to obtain tactile sensors with high performance, high resolution, high sensitivity, and rapid response for various applications. In this section, we present a brief introduction to the structural design and functional exploration of flexible pressure sensors from different perspectives.

### 3.1 Strategies for Achieving Flexible and Stretchable E‐Skin

Compared with the traditional electronic devices that are fabricated on rigid semiconductor wafers, flexible and stretchable electronics are a topic of growing interest as alternative future electronics because of their potential applications in micro‐electromechanical systems (MEMS), robotics, human–machine interactions, and biological tissue sensing.^38^ Many flexible electronics have been demonstrated by sufficiently reducing the thickness of the substrates to acquire remarkable bendability; however, this approach is limited to nearly flat substrates.^39^ In contrast, stretchable electronics can conformally adhere to complicated and uneven surfaces similar to human skin. However, the achievement of stretchable e‐skin is more difficult than the construction of a flexible device from the perspective of preparation technology.^40^

**Figure 3:**
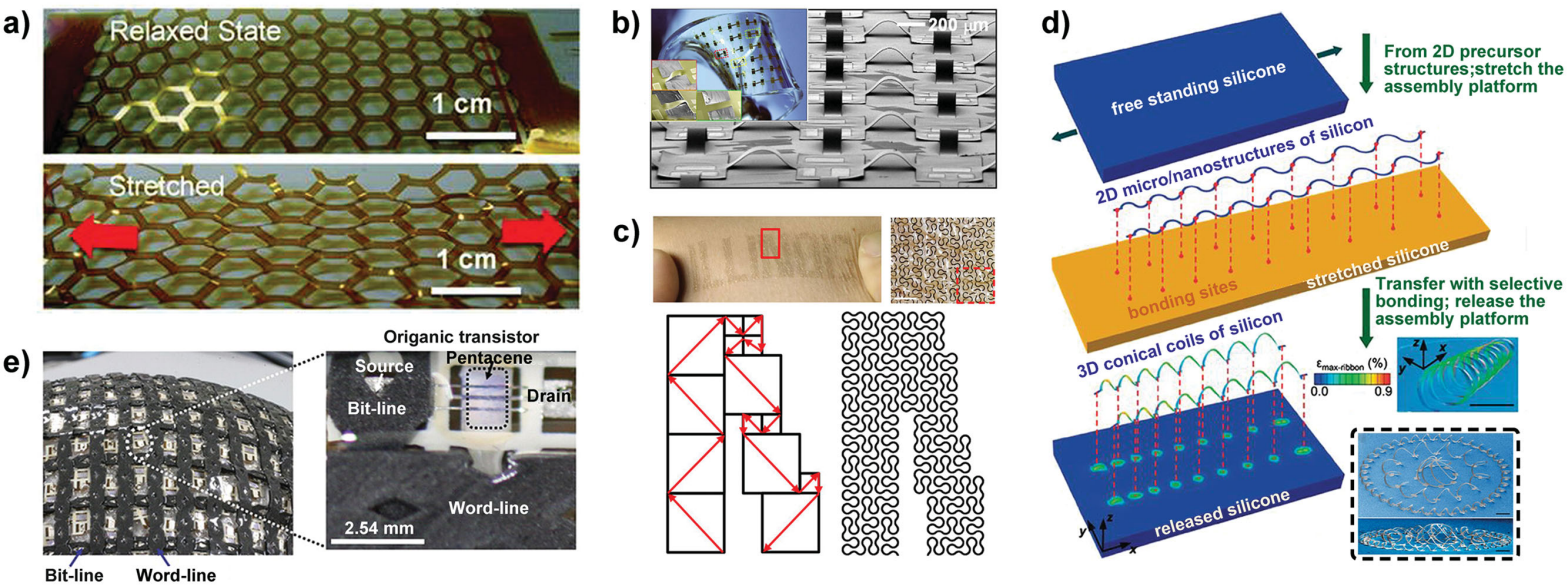
Different strategies to achieve stretchability. a) Net‐shaped structural design. Reproduced with permission.[[qv: 41b]] Copyright 2011, American Chemical Society. b) Noncoplanar mesh design. Reproduced with permission.^44^ Copyright 2008, National Academy of Sciences. c) Fractal design. Reproduced with permission.^45^ Copyright 2014, Macmillan Publishers Ltd. d) 3D architectures via compressive buckling. Reproduced with permission.^47^ Copyright 2015, American Association for the Advancement of Science. e) Use of elastic conductors. Reproduced with permission.^49^ Copyright 2008, American Association for the Advancement of Science.

To date, two common strategies have been used to improve the stretchability of the devices. The first method directly bonds thin conductive materials that have low Young's moduli to a rubber/elastic substrate, such as poly(dimethylsiloxane) (PDMS). Geometrical patterning and device designs, e.g., net‐shaped structures,^41^ were also employed to further enhance stretchability and adaptability (**Figure** **3**a). Rogers and co‐workers has pioneered many strategies for the adherence of conventional inorganic materials with excellent electronic performance and rigidity to elastomeric soft substrates.^42^ Inorganic semiconductors, including electronic components and interconnectors, were assembled into stretchable devices using neutral mechanical plane layouts to ensure that the strains of the high‐moduli materials are negligible using a complex wavy structure that can absorb the major tensile strains formed in the process of stress release of soft substrates.[[qv: 9a]],^43^ The island‐bridge design was first presented to considerably improve the stretchability; in this design, active components with high effective stiffness act as floating islands, and interconnectors with low effective stiffness serve as tensile bridges (Figure 3b).^44^ The non‐coplanar structures associated with deformable interconnecting structures, including straight ribbons and serpentine traces, allowed the device to undergo complex deformations, such as rotation and twisting. Recently, fractal layouts with self‐similarity served as alternative interconnectors, such as Peano and Vicsek curves, were proposed for stretchable devices for further adaption to various deformations (Figure 3c).^45^ Three types of contact modes between the interconnector and the soft substrate were employed, including nonbonded, partially bonded, and completely bonded contacts, to accommodate the practical demands required by different applications. For example, devices with nonbonded designs deliver the best mechanical properties due to their free deformation capabilities but are more susceptible to physical damage owing to the exposure of interconnectors. Fortunately, a novel method using dielectric microfluidics with suitable viscosities was developed to extend the service duration of the device compared with that of solid encapsulation.^46^ In addition, complex and varied 3D structures, e.g., flowers, spherical baskets and fences, were further reported in the context of compressive buckling and were shown to have widespread potential applicability in MEMS, 3D electronics, and biological systems (Figure 3d).^47^

The second method to further enhance the stretchability of electronics is to assemble the device using intrinsically stretchable conductors that are always fabricated by mixing conductive materials into an elastomeric matrix.^48^ Someya and co‐workers demonstrated a conformal 3D active matrix transistor with a highly elastic composite film consisting of uniformly distributed carbon nanotubes in a fluorinated copolymer, which was facilitated by the use of an ionic liquid (Figure 3e).^49^ The electrical performance of the stretchable electronic circuits remained unchanged under a 70% biaxial tensile strain. Another conductive and printable polymer composite composed of multi‐walled carbon nanotubes and silver was synthesized by Chun et al., and the conductivity could maintain a value of 20 S cm^−1^ under stretching to 140%.^26^ Park et al. also developed a conductive circuit, with a conductivity of up to 2200 S cm^−1^ at 100% strain, based on rubber fibers covered with silver nanoparticles via electrospun technology for large‐scale production.^50^ Recently, Bao and co‐workers successfully created a stretchable transistor that retained its transistor characteristics at up to 250% strain using the stress adaptability of microcracks.^51^ The key technical challenge lies in achieving an optimal “trade‐off” between electrical conductivity and mechanical elasticity because the increase in conductivity is typically achieved at a price, namely, a decrease in elasticity when the ratio of the conductive composition in the elastic matrix is increased.

### 3.2 Large‐Scale E‐Skin Based on Active Transistor Arrays for Pressure Mapping

Large‐scale pressure sensor arrays are important for the application of future generations of e‐skin.^52^ However, the signal crosstalk of tactile sensors based on the resistance or capacitance transduction mechanisms always results in inaccurate measurements, thus presenting one of the greatest challenges to the development of e‐skin. Fortunately, the use of transistors provides an opportunity to reduce crosstalk between pixels with rapid addressing and low‐power consumption due to its perfect functionality of signal transduction and amplification as an ideal electronic component. Hence, many studies on transistor arrays have been performed to obtain large‐scale and flexible pressure sensor arrays for intelligent artificial e‐skin and wearable devices.[[qv: 6a]],^23^, ^53^, A series of fabrication techniques has been developed, including photolithography and printing processes,[[qv: 14b]],^54^ and different types of active channel materials have been investigated, including inorganic crystalline semiconductors,[[qv: 15b]] organics,^55^ graphene,^56^ NWs,^57^ and carbon nanotubes,[[qv: 52a]],^58^ each of which offers distinct advantages in the characteristic transistor parameters, such as carrier mobility, operating voltage, and on/off current ratio. For example, the organic field‐effect transistor (OFET) exhibits higher flexibility and lower preparation cost but has lower carrier mobility and larger operating voltages compared with those constructed of inorganic semiconductors. Recently, Bao and co‐workers designed logic circuits with higher noise margins based on n‐type and p‐type carbon nanotube transistors that can be used to appropriately tune the threshold voltage by optimizing the dopant thickness or concentration.^59^ In addition, to render the transistor more stretchable and flexible, several previously mentioned strategies, such as island‐bridge layouts and network structures, are also feasible for the fabrication of active transistor arrays.[[qv: 41b]]

**Figure 4:**
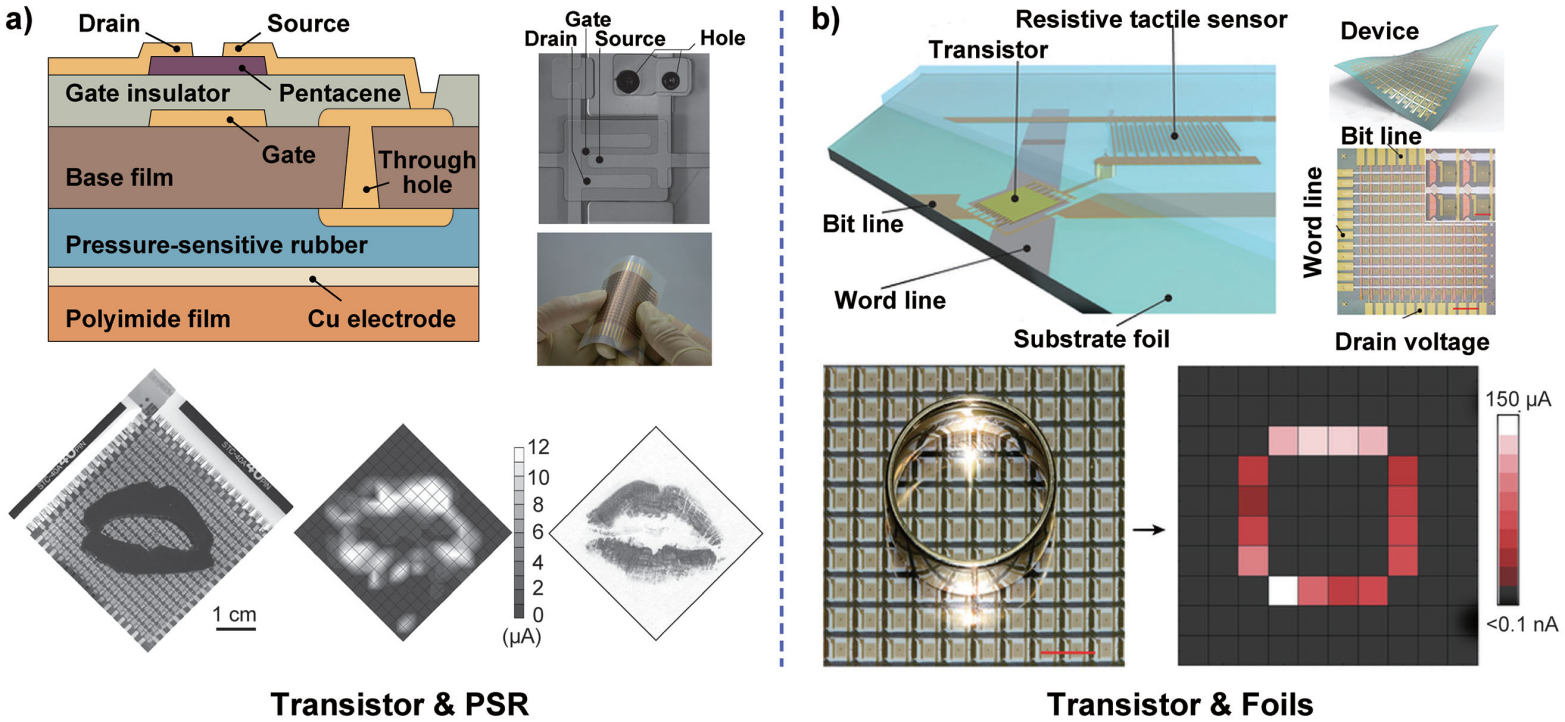
Two typical structure diagrams of pressure‐sensitive transistor active matrix sensors based on the piezoresistive transduction methods. a) Transistor array laminated with a pressure‐sensitive rubber layer. Reproduced with permission.^23^ Copyright 2004, National Academy of Sciences. b) Transistor array integrated with resistive tactile sensing foils. Reproduced with permission.^12^ Copyright 2013, Macmillan Publishers Ltd.

**Figure 5:**
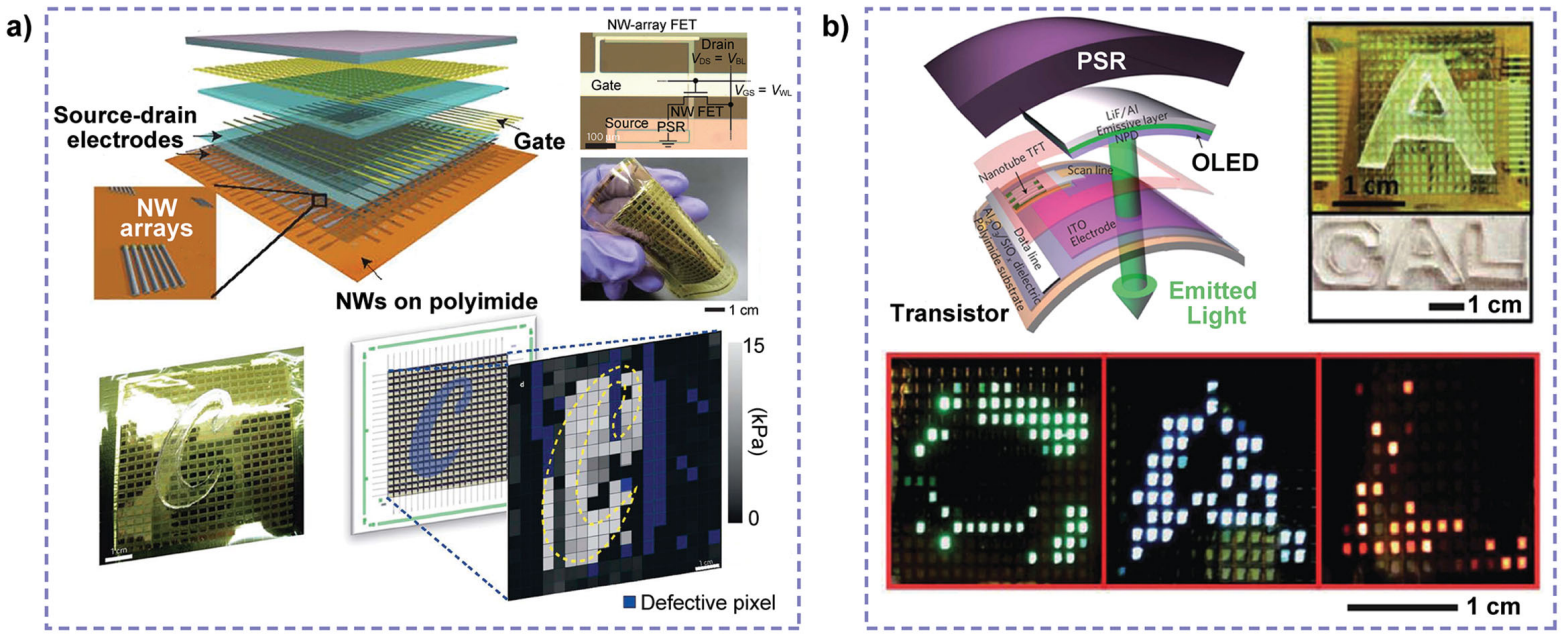
Transistor active matrix sensors for pressure mapping. a) Ge/Si nanowire‐array FETs integrated with a PSR sheet. Reproduced with permission.^57^ Copyright 2010, Macmillan Publishers Ltd. b) Carbon nanotube TFTs integrated with a PSR layer and OLEDs to achieve pressure visualization. Reproduced with permission.^61^ Copyright 2013, Macmillan Publishers Ltd.

The excellent electronic switching action of the transistor first attracted widespread interest to exploit pressure‐sensitive sensors for applications in intelligent e‐skin. Someya et al. pioneered the use of an OFET active matrix laminated with a PSR layer to serve as a high‐performance pressure sensor array (**Figure** **4**a).^23^ The transistor was used for rapid addressing with low‐power consumption, of which gate electrodes and drain electrodes are connected to a word line and a bit line, respectively and source electrodes are linked to the ground through the PSR. Resistance of the PSR sheet used as sensing element changes with different pressure levels, resulting in variation of the transistor's gate voltage, leading to a change in the drain current. A large‐scale pressure image was thus obtained by using a 16 × 16 pixelated transistor matrix with a resolution of 10 dpi. This type of device design, i.e., a pressure‐sensing element and a switching matrix, allows the flexible organic transistor to have wide application prospects in artificial intelligent e‐skin. By embedding a floating gate into the gate dielectric, a pressure‐sensitive nonvolatile memory transistor was created that can store or erase the distribution of pressure for long durations by charging or discharging the floating gate.^60^ Moreover, instead of a rubber sheet, the use of resistive tactile sensing foils based on the change in surface conductivity further improved the resolution and reduced the signal crosstalk between pixels,^12^ which also allowed the device to become thinner (2 μm total thickness) and lighter (3 g m^−2^) with ultra‐flexible mechanical properties and remarkable electrical properties (Figure 4b). Additionally, considering the advantage of inorganic semiconductors with high carrier mobility, Javey and co‐workers successfully demonstrated a low‐power transistor active matrix using parallel Ge/Si nanowire arrays that operated at a low voltage (**Figure** **5**a).^57^ To realize the visual pressure display, a user‐interactive e‐skin based on the flexible pressure sensor was also developed.^61^ The organic light‐emitting diodes were laminated between the transistor active matrix and the PSR layer, which could be turned on with a decrease in the rubber's resistance via an applied pressure (Figure 5b). The luminance intensity of each pixel depends on the pressure, thus providing visualized mapping of the pressure profile. The device can also monitor the drain current of the transistor and is promising for broad applications in human–machine interaction systems, smart wallpapers, intelligent e‐skin, and health monitoring. However, the pressure sensitivity and response time of these devices are limited by the laminated materials of the pressure‐sensing element.

**Figure 6:**
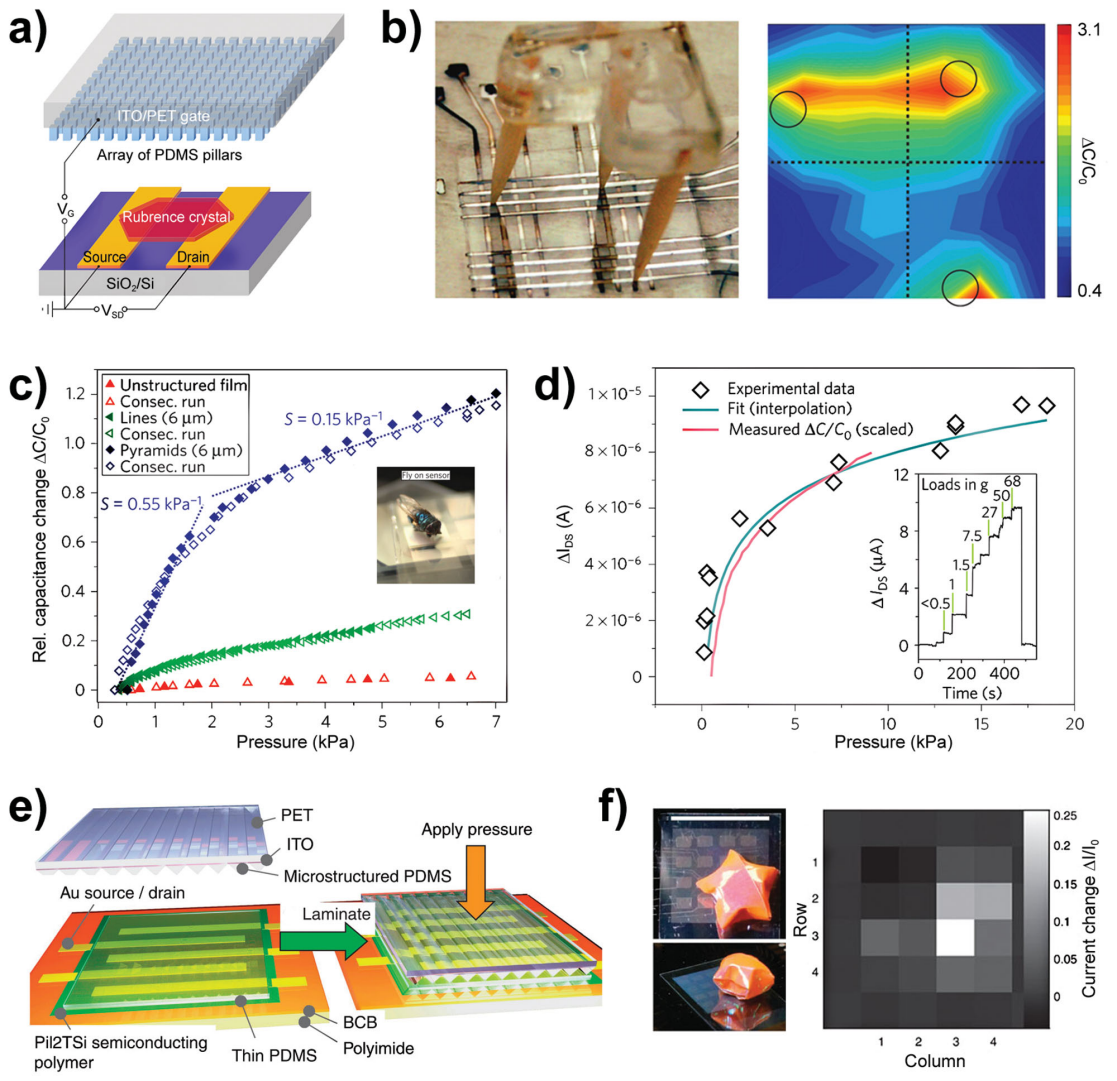
Highly sensitive active matrix sensors with geometric design of gate dielectric. a) Pressure‐sensitive OFET with microstructured PDMS dielectric layer. b) Flexible pixel‐type capacitive pressure sensor array using a microstructured PDMS film as a dielectric layer. c) Pressure response curves of the capacitive sensors fabricated with different types of microstructured PDMS films. d) Change in *I*_DS_ of the OFET in response to pressure, which is proportional to the change in relative capacitive and exhibits a rapid response. Reproduced with permission.^31^ Copyright 2010, Macmillan Publishers Ltd. e) Flexible active matrix with high‐pressure sensitivity and a microstructured dielectric layer. f) 2D pressure imaging using a transistor active matrix. Reproduced with permission.^62^ Copyright 2013, Macmillan Publishers Ltd.

Therefore, a new strategy used to obtain active matrixes with high‐pressure sensitivity that reach the lower regimes of the measured pressure range and have shorter response times is urgently required to meet the demands of additional applications. Bao and co‐workers successfully integrated a novel type of high‐pressure‐sensitive organic transistor with a microstructured compressible gate dielectric on a rigid Si wafer (**Figure** **6**a,b).^31^ The capacitance‐dependence pressure sensitivity of the pyramid‐structured PDMS was greatly enhanced compared with unstructured or other different microstructured films because the increasing additional air voids between the PDMS dielectric layer and the organic semiconductor have lower dielectric constants, which was determined according to the field‐effect transistor theory, as follows:(1)
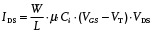
where *I*_DS_ and *V*_DS_ are the drain/source current and voltage, respectively, *W* and *L* are the channel width and length, respectively, *μ* is the mobility, *C_i_* is the specific gate capacitance, *V*_GS_ is the gate voltage, and *V*_T_ is the threshold voltage; it is clear that *I*_DS_ is proportional to the specific gate capacitance. Therefore, the drain current of the transistor can respond quickly to the change in the applied pressure, thus allowing high sensitivity of the device in the low‐pressure regime (less than 2 kPa) (Figure 6c,d). A further improvement was shown to demonstrate a flexible, pressure‐sensitive active matrix on a plastic substrate with high‐pressure sensitivity and rapid response time based on microstructured rubber, which could be used to precisely map the static pressure distribution and monitor health conditions (Figure 6e,f).^62^ Recently, Zang et al. developed another novel structure with suspended gate electrodes by directly adding an air dielectric layer instead of the previous structured elastic dielectric layer. This flexible device can detect lower pressures of 0.5 Pa with notably low‐power consumption, which offers particular advantages in the sensing of ultra‐low pressures, such as sound pressure.^30^

### 3.3 High‐Resolution E‐Skin Based on Piezotronic/Piezo‐Phototronic Effect

To fabricate high‐resolution flexible pressure sensor arrays, each pixilated sensor unit should be miniaturized to dimensions that are as small as possible. Compared with piezoresistive or capacitive sensors for which the quality of the output signals will significantly decrease with the size of the device, piezoelectric sensors are more adaptable to miniaturization due to the reduced size dependency of piezopotential signals. Wang's group has continuously constructed subminiature piezoelectric sensors based on the piezotronic effect using NWs or NBs.^8^ Micro/nanosized flexible strain/force sensors,^63^ strain‐gated logic devices,^64^ and other flexible electronics^65^ have been successfully developed using inner‐crystal piezopotentials to tune the charge carrier transport properties in certain piezoelectric semiconducting NWs or NBs (ZnO,^66^ GaN,^67^ CdS,^68^ and CdSe,^69^ among others.^70^ Furthermore, by introducing photo excitation/emission into piezotronics, piezo‐phototronics were proposed, which operate by tuning/controlling the electro‐optical processes via piezoelectricity,^71^ thus providing the possibility of acquiring additional high‐resolution pressure mapping by converting mechanical stimuli into optical signals. Recently, piezotronic and piezo‐phototronic pressure sensors were integrated into high‐resolution arrays using micro/nanofabrication technologies, thereby promoting the realization of high‐resolution pressure mapping for tactile emulation in e‐skin.^72^

**Figure 7:**
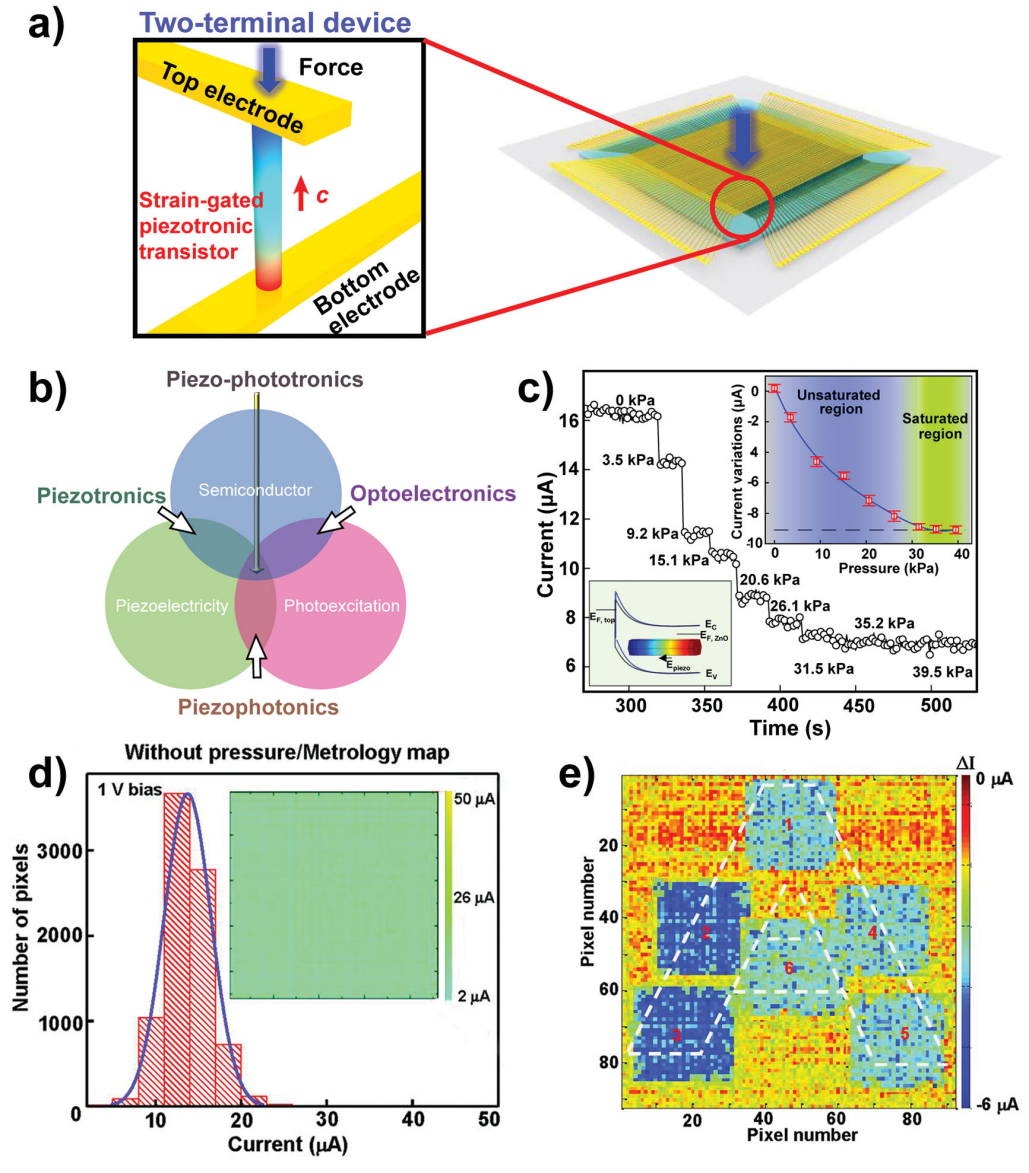
Pressure mapping based on piezotronics. a) Schematic structure of a 3D strain‐gated vertical piezotronic transistor matrix. The color gradient in the enlarged view inset illustrates the piezopotential field induced by the applied force, in which red and blue, respectively, represent positive and negative piezopotentials. b) Origins of piezotronics, piezo‐phototronics, and piezophotonics, which provide the coupling between/among the semiconductor, piezoelectricity, and photoexcitation. c) Current response of a piezotronic transistor to applied pressure. d) Statistical current distribution of the integrated transistor array without applied pressure. e) Current distribution of the array under pressure. Reproduced with permission.[[qv: 15b]] Copyright 2013, American Association for the Advancement of Science.

To realize high‐resolution tactile mapping, Wu et al. demonstrated a highly integrated 3D strain‐gated vertical piezotronic transistor matrix consisting of a 92 × 92 tactile pixel array in an area of 1 cm^2^ on a flexible substrate (**Figure** **7**).[[qv: 15b]] Each pixel consists of one or more vertically grown ZnO NWs electrically connected to the bottom and top electrodes, thus forming two Schottky contacts. The variance of the piezopotential in the ZnO NWs with applied force serves as the gate voltage to control the local Schottky barrier heights, controlling the transport characteristics to enable the stain‐gated two‐terminal transistor that can detect pressure. By plotting the current variation of each pixel before and after the application of pressure, pressure mapping was obtained from which not only the coordinate but also the value of applied pressure could be determined (Figure 7d,e). A shape‐adaptive pressure‐sensing test was performed to demonstrate the application of the piezotronic matrix as e‐skin to mimic the human sense of touch. However, the response time of the piezotronic transistor was measured as ≈0.15 s, which hinders the device from providing instant perception of tactile stimulation.

**Figure 8:**
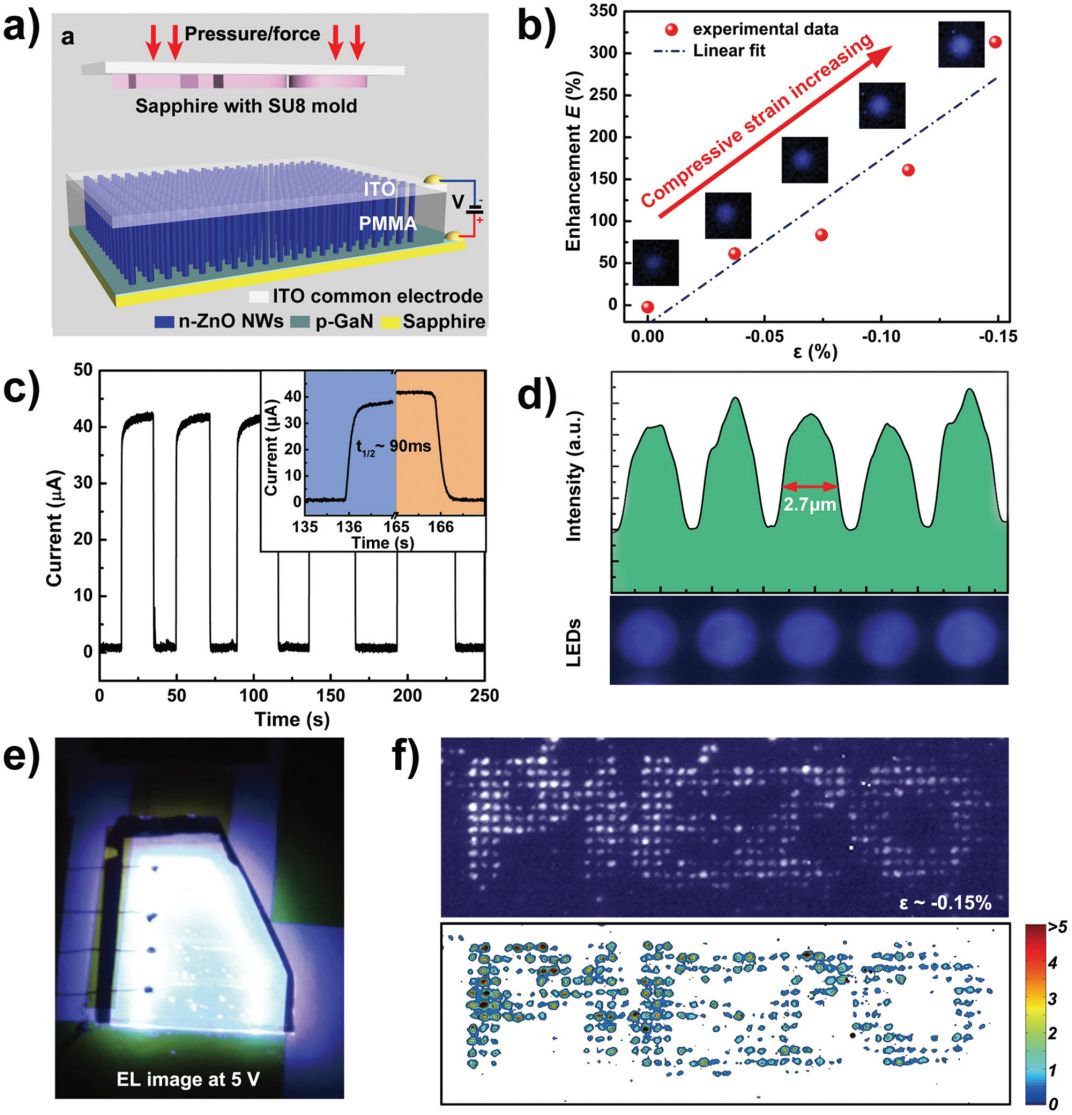
Pressure mapping based on piezo‐phototronics. a) Schematic structure of the nanowire‐LED‐based pressure sensor array. b) Change in emission enhancement of an LED pixel in response to strain. c) Measured response time of the device is ≈90 ms. d) Spatial resolution of the array is estimated as 2.7 μm. e) Photograph of a electroluminescent device under a bias voltage of 5 V. f) High‐resolution pressure imaging. Reproduced with permission.[[qv: 15a]] Copyright 2013, Macmillan Publishers Ltd.

**Figure 9:**
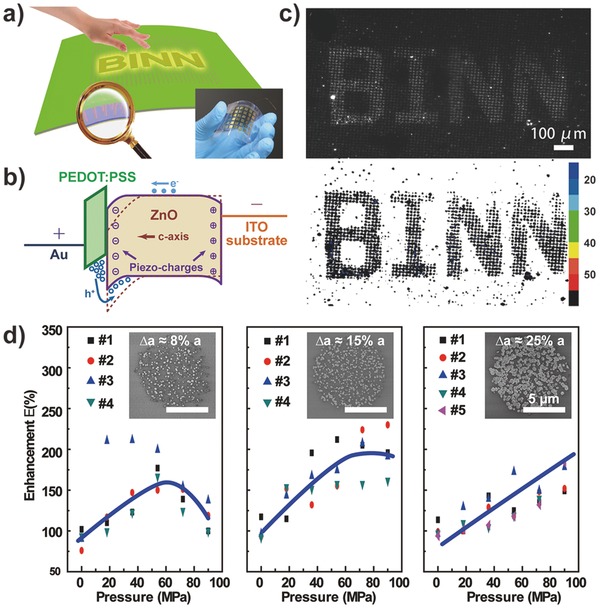
Flexible piezo‐phototronic nanowire LED array for pressure mapping. a) Schematic illustration and photograph of the flexible device. b) Electron band diagram showing the piezo‐phototropic effect. c) Pressure mapping result. d) Light intensity enhancement as a function of the area of ZnO NWs. Reproduced with permission.[[qv: 73a]]

To improve the response speed for tactile mapping, our group reported an effective solution using the piezo‐phototronic effect in a nanowire‐LED array to obtain a response time of ≈90 ms, a value that is primarily limited by the pressure loading rate (**Figure** **8**).[[qv: 15a]] In this device, a p‐GaN thin film and a highly ordered n‐ZnO NW array form p‐n junctions in which each nanowire becomes an individual light emitter under the appropriate forward bias voltage. The piezoelectric polarization charges of ZnO nanowire were introduced by compressive pressure, resulting in a local dip in the energy band at the junction region. The distorted band tends to trap holes near the junctions, enhancing the carrier injection and recombination rates, hence increasing the emitting intensity under the piezo‐phototronic effect. The emission intensity of the pixels under compression is enhanced dramatically and nearly linearly with the compressive strain, whereas the pixels that are not compressed show no evident change when pressure is applied to the sensor array through a convex character pattern of “PIEZO,” producing a quantified image of applied pressure via an electroluminescence contour map (Figure 8f). Note that the time required for the entire mapping process is independent of the detection area because the output optical signals from all pixels are read out in parallel by a digital camera, revealing the promising application of this method for future generations of optical‐communication‐based e‐skins and other intelligent human–machine interactions. Based on this study, we further developed a flexible LED pressure mapping matrix consisting of an organic p‐PEDOT:PSS (poly (3,4‐ethylenedioxythiophene)–polystyrenesulfonic acid) film and patterned n‐ZnO NWs on a flexible substrate (**Figure** **9**).^73^ The device can still operate normally after long periods of bend‐and‐release, revealing the potential applications of the piezo‐phototronic matrix in practical e‐skin.

**Figure 10:**
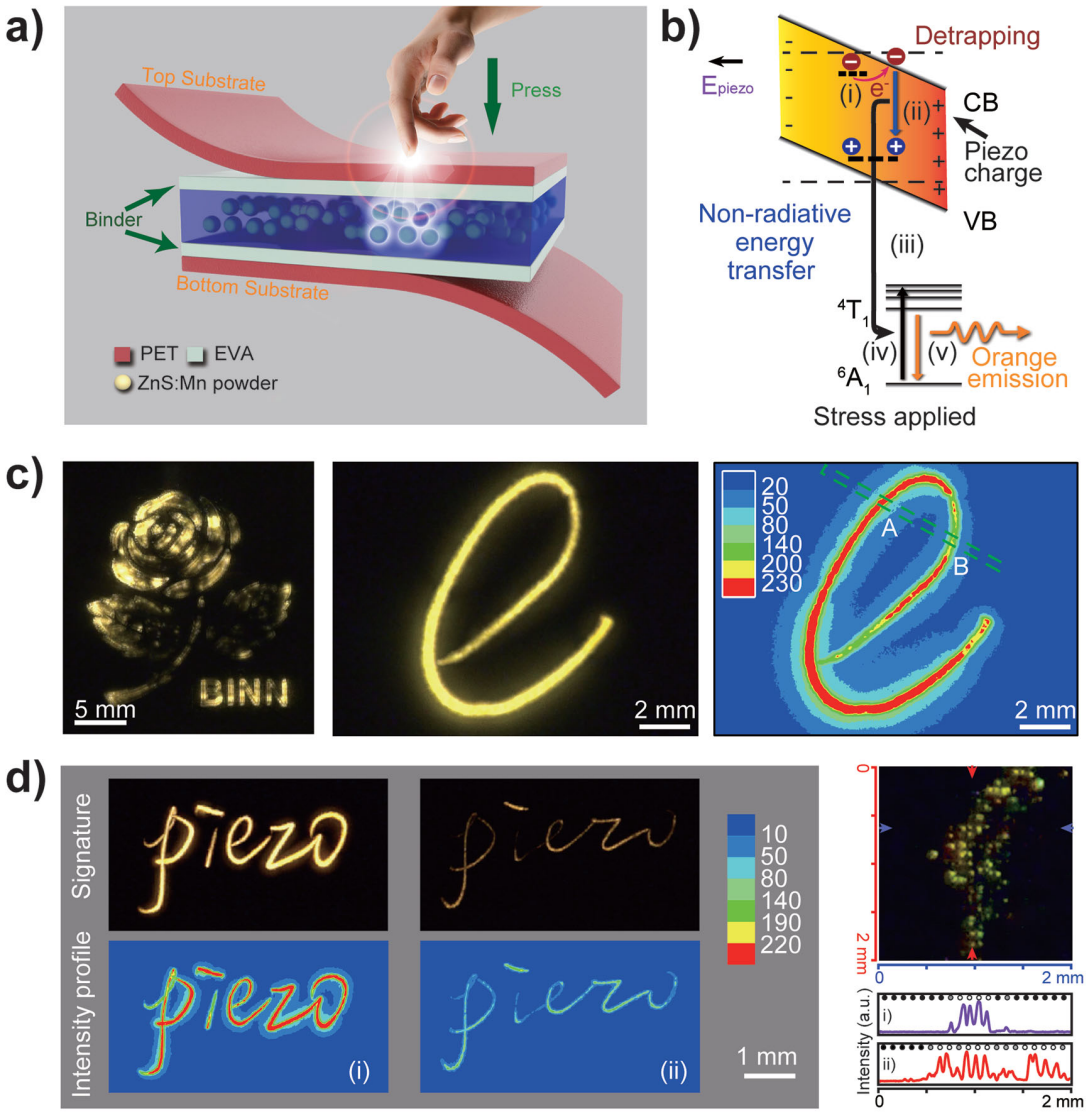
Pressure mapping based on piezophotonics. a) Schematic structure of the device. b) Band diagram of ZnS:Mn, which indicates the action mechanism of piezophotonic effect. c) Dynamic pressure mapping of 2D planar and single‐point models. d) Signature recording and high‐resolution pressure mapping. Reproduced with permission.^74^

A rapid‐response flexible pressure sensor matrix based on the direct conversion of mechanical stress into an optical signal was developed using the piezophotonic property of ZnS:Mn particles (**Figure** **10**).^74^ The core of the piezophotonic property is a piezoelectric‐induced photon‐emission process. The electron energy band of piezoelectric ZnS is tilted by the piezopotential under pressure, which can promote the excitation of Mn^2+^ ions, and the following de‐excitation gives rise to photon emission in the form of yellow light with an intensity positively correlated to the applied pressure. A rapid response time of less than 10 ms was obtained in this stress‐to‐light conversion process, and a spatial resolution of 100 μm was realized using photolithography; this approach could be further improved with other micromanufacturing engineering and technology advances. Using an image acquisition and processing system constructed in the lab, the sensor matrix could record a single‐point sliding dynamic pressure in handwriting for signee identification and could map a 2D planar pressure distribution by extracting the emission intensity plot in real time (Figure 10c). All of these features render this piezophotonic device one of the most promising candidates for rapid‐response and high‐resolution pressure‐mapping e‐skin systems.

## 4 Recent Developments in Novel E‐Skin

In addition to basic pressure‐sensing ability, e‐skins are endowed with an increasing amount of additional properties for practical and promising applications. Specifically, the self‐powered capability is exploited in e‐skins for artificial intelligence systems that must continuously operate for long durations. Multifunctional integration that includes self‐healing ability, multi‐stimuli differentiation, and simultaneous temperature and pressure sensing is intended to imitate or even surpass the sensory ability of human skin. In addition, wireless technology is combined with e‐skins for convenient data and energy transmission. In this study, we summarize these recent developments in the following section.

### 4.1 Self‐Powered E‐Skin

The power consumption of pressure/tactile sensors is a significant obstacle for integrated and practical e‐skin systems. Different strategies, such as the design of the appropriate resistance and operating voltage for piezoresistive sensors^21^ and the adoption of thin dielectric materials with high dielectric constants in capacitive/OFET‐based sensors,^62^ are considered effective approaches to reduce power consumption.^53^, ^75^ An alternative and ultimate approach used to settle the power consumption issues in e‐skin is the fabrication of self‐powered sensors.^76^ Therefore, studies on flexible or stretchable solar cells,^77^ piezoelectric nanogenerators,^78^ and triboelectric generators or sensors^79^ have attracted increasing attention in recent years. Among these research topics, the developments in triboelectric generator/sensor are dramatically growing due to their facile fabrication, self‐powered ability, low cost, and diverse applications.^80^

**Figure 11:**
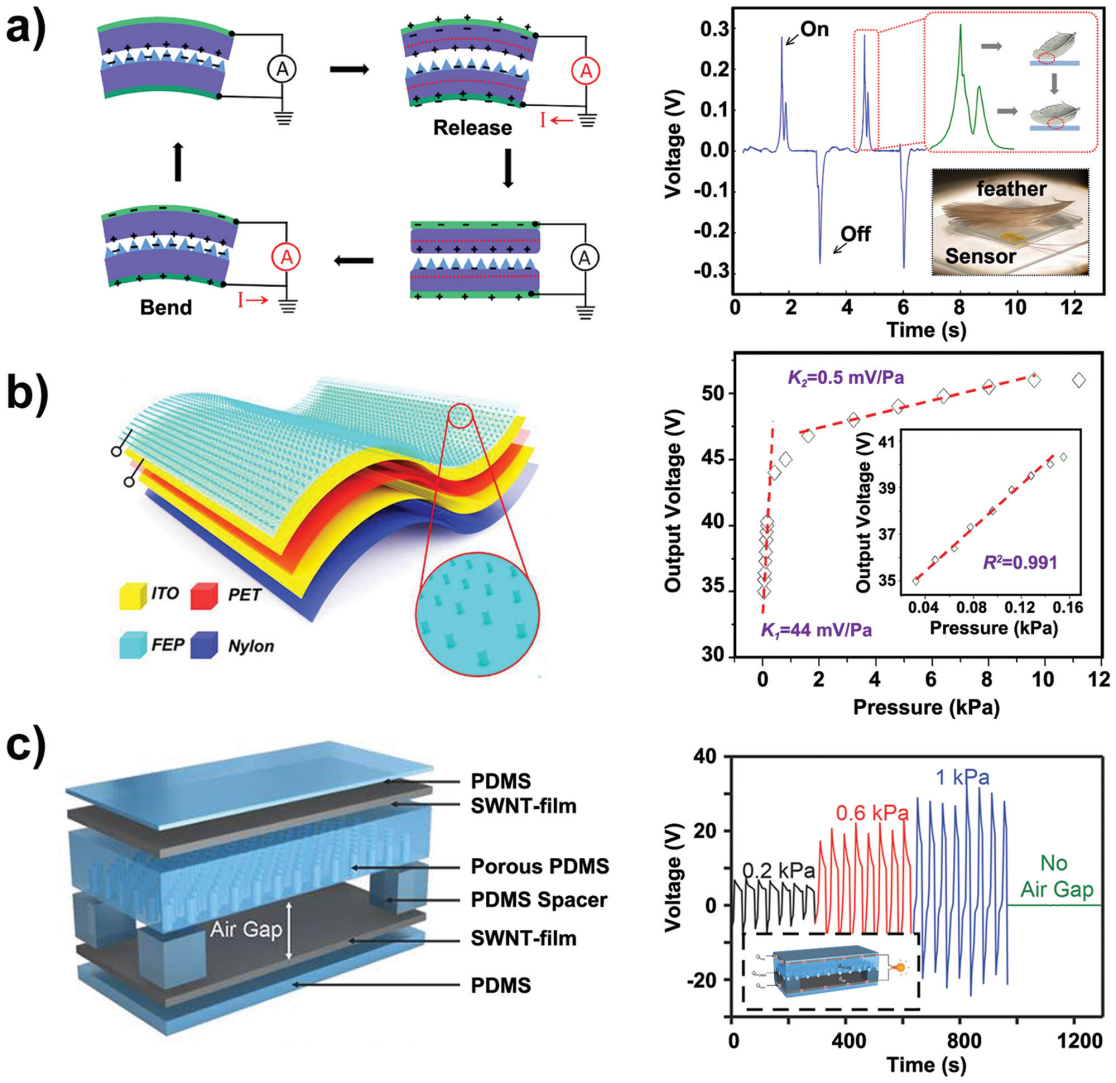
Developments in self‐powered pressure‐sensitive triboelectric sensors. a) First flexible triboelectric self‐powered pressure sensor. Reproduced with permission.^81^ Copyright 2012, American Chemical Society. b) Self‐powered ultra‐sensitive flexible tactile sensor. Reproduced with permission.^82^ Copyright 2014, American Chemical Society. c) Stretchable tactile sensor that can differentiate multiple mechanical stimuli and could be self‐powered. Reproduced with permission.^83^

The first flexible triboelectric generator that could convert random mechanical energy into electrical signals (voltage/current) without power consumption was reported by Fan et al.[[qv: 16b]] The basic operating mechanism of this device can be explained as follows. Charges transfer between the contact surfaces of two stacked polymer films due to their friction under mechanical stimuli resulting from the triboelectric effect, thus introducing an electric potential difference between the top and bottom electrodes deposited on the outer surfaces of the films because of electrostatic induction; hence, electrical signals are detected when the two electrodes are connected through a electrometer. By constructing microstructures on the contact surfaces, the pressure sensitivity of the flexible triboelectric generators was improved such that they could operate as self‐powered pressure sensors (**Figure** **11**a).^81^ For example, Zhu et al. designed a type of ultrasensitive tactile sensor by employing the nanowire‐structured surface of highly triboelectric‐negative fluorinated ethylene propylene (FEP) film as the contact surface (Figure 11b).^82^ In addition, Bao and co‐workers combined triboelectricity generation with capacitive pressure sensing and resistive lateral strain sensing for multiple mechanical stimuli differentiation (Figure 11c), enabling highly sensitive pressure detection by constructing an air gap between a porous PDMS film and an SWCNT film.^83^

**Figure 12:**
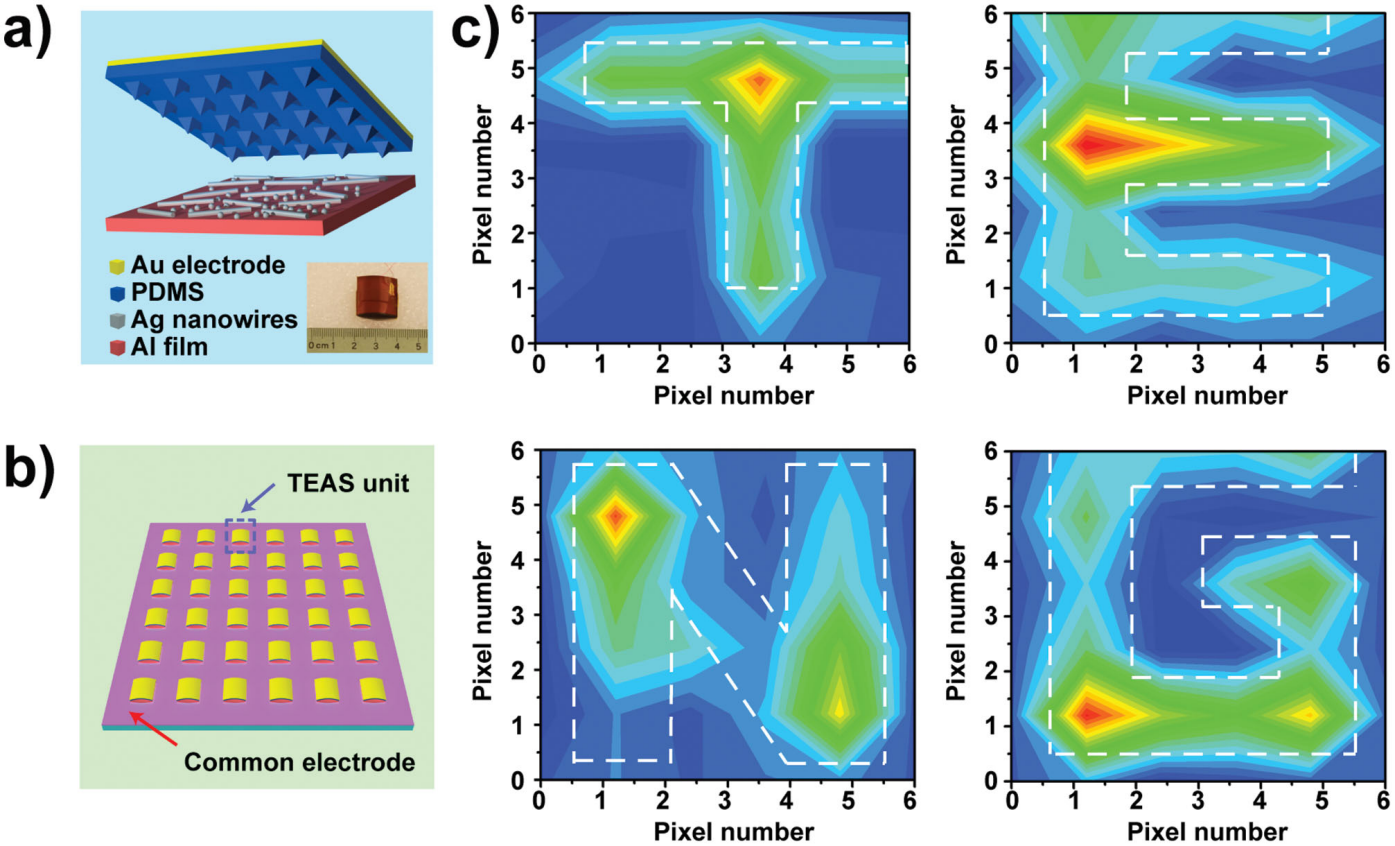
Integrated self‐powered triboelectric sensor array capable of mapping applied pressure. a) Structural illustration of a single triboelectric sensor with a photograph of the device as the inset. b) Schematic illustration of an integrated triboelectric sensor array. c) The 2D output voltage contour plots from the sensor matrix under external pressure applied through architectures with designed calligraphy. Reproduced with permission.^84^ Copyright 2013, American Chemical Society.

**Figure 13:**
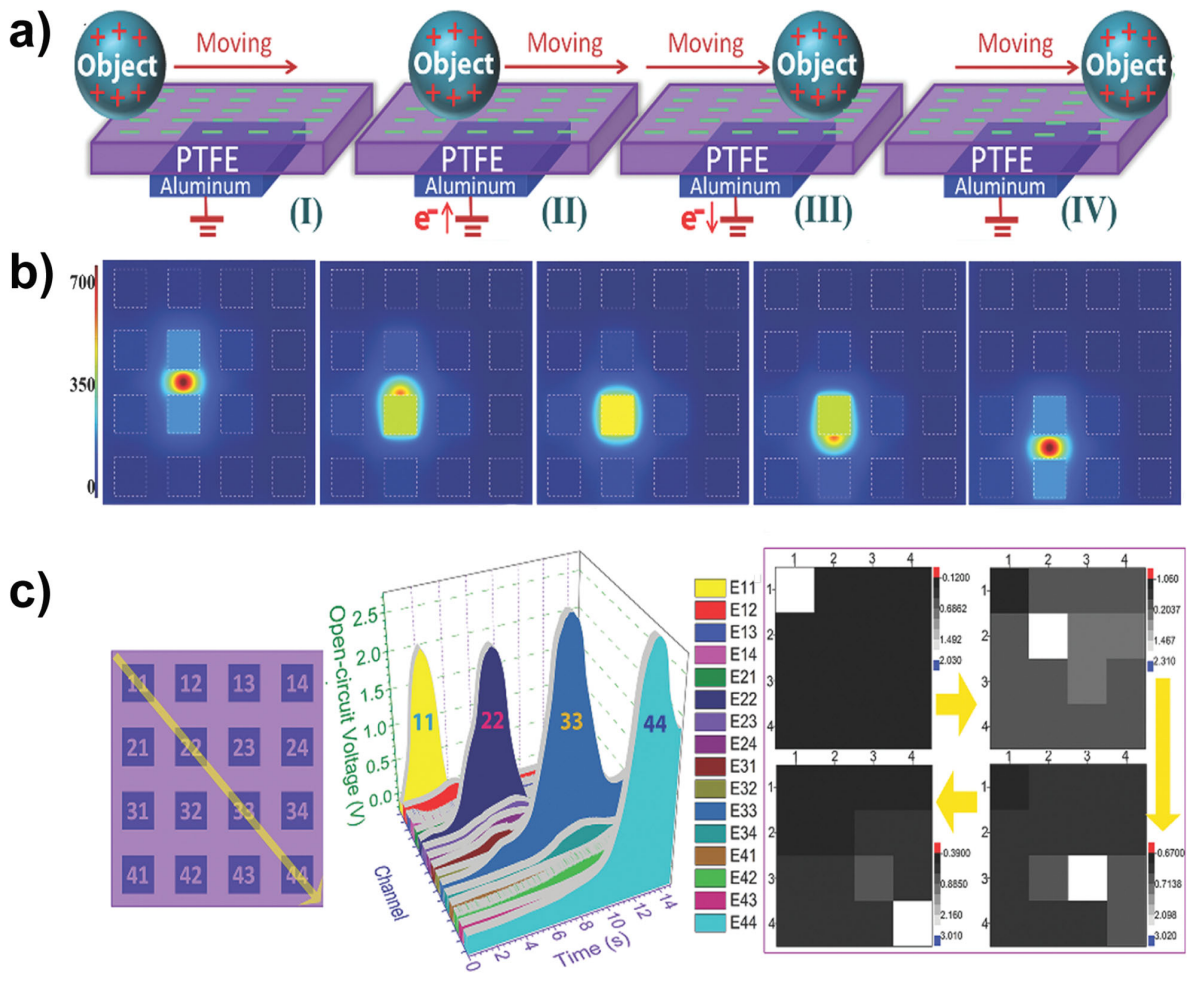
Self‐powered single‐electrode‐based triboelectric sensor array that can track the motion trajectory of an object. a) Schematic diagram of the device and its working process of the sensor array. b) Finite element simulation of the potential distribution on the array when a ball moves on the surface of the PTFE film. c) Motion imaging result of an Al ball moving on the sensor array along a specific path. Reproduced with permission.^86^

The integration of a triboelectric sensor array was reported by Lin et al. for self‐powered tactile imaging (**Figure** **12**).^84^ An array consisting of discrete triboelectric sensor units was assembled to provide mapping of applied pressure using the output voltage contour plot from all units. This integration method is not suitable for the fabrication of large‐scale high‐resolution arrays with numerous pixels because each pixel is independent in this array. The development of single‐electrode‐based triboelectric sensors provides a more suitable integration method.^85^ Yi et al. demonstrated self‐powered motion tracking through a triboelectric sensor that consisted of arrayed aluminum electrodes beneath a piece of polytetrafluoroethylene (PTFE) film. A voltage peak is detected from an Al electrode due to the electrostatic‐induced potential variation between the electrode and the electrical ground when a sensed object with triboelectric charges moves to the point over the center of the electrode, thus providing information about the motion trajectory as well as the moving velocity and acceleration data, revealing the possibility for large‐scale integration of triboelectric sensors in a facile manner (**Figure** **13**).^86^ Han et al. realized the integration of a high‐resolution array using the weave technique to fabricate a single‐electrode‐based self‐powered trajectory tracking array.^87^ These studies, along with related works, have demonstrated the usability of the triboelectric devices in applications, such as self‐powered wearable electronics,^88^ healthcare monitoring, and self‐powered e‐skin systems.^89^

### 4.2 Multifunctional E‐Skin

The ability to mimic the complex function of human skin is of tremendous interest in the development of artificial e‐skin. Functions in addition to pressure or tactile sensing are desirable to better adapt to the surrounding environment, including the abilities of self‐healing and temperature measurement, which are also key challenges for practical applications of e‐skin.

**Figure 14:**
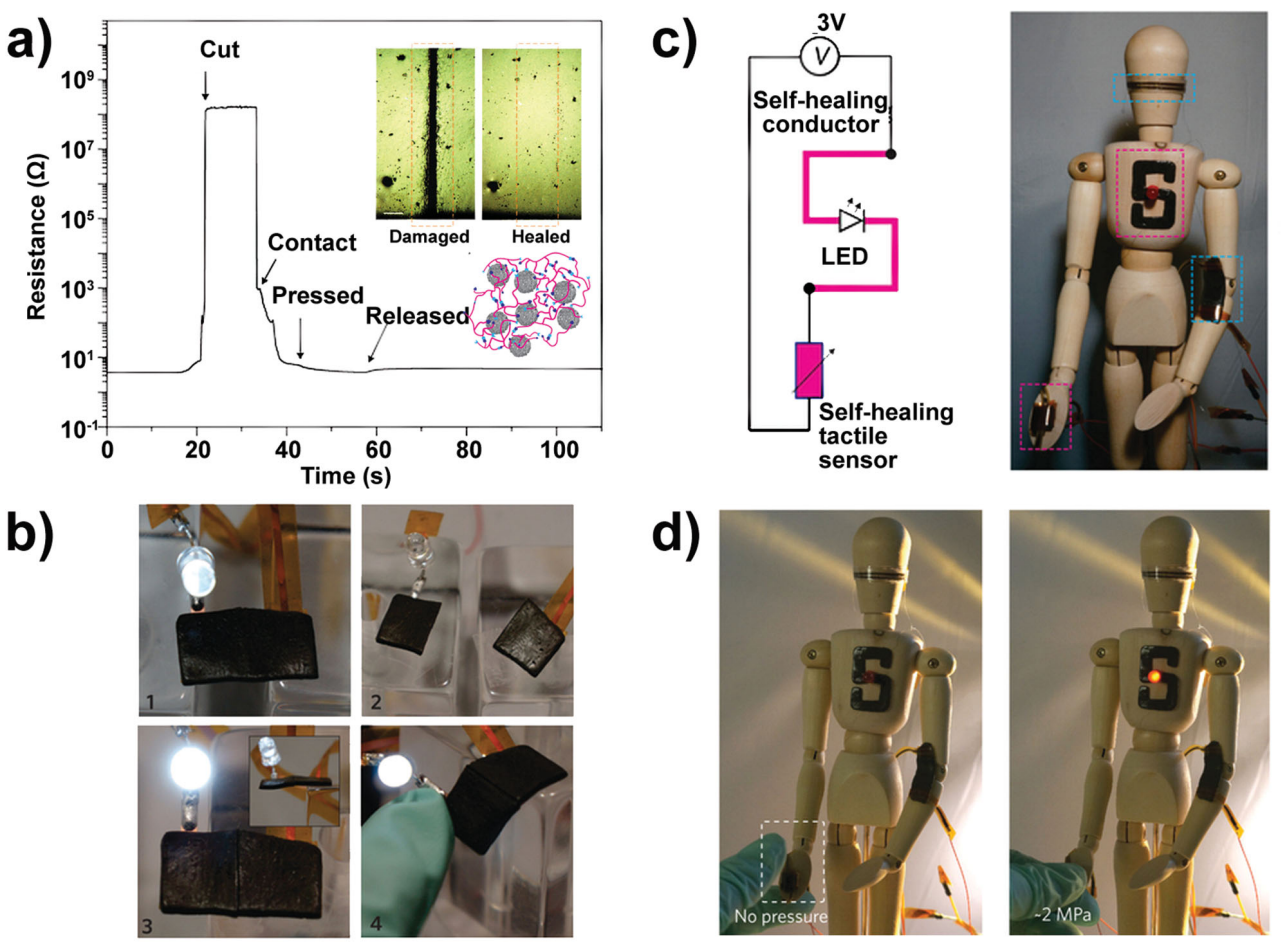
Self‐healing pressure sensor for e‐skin. a) Electrical healing process recorded by the time evolution of measured resistance. Inset: Optical images of damaged and self‐healed samples (upper) and proposed interaction of oligomer chains with Ni particles (lower). b) Demonstration of the self‐healing process of a conductive composite using a LED. c) Circuit diagram and photograph of a self‐healing tactile sensor mounted on a doll. d) Demonstration of self‐healing process in electrical and mechanical characterization. Reproduced with permission.[[qv: 11a]] Copyright 2012, Macmillan Publishers Ltd.

The self‐healing property has currently attracted profound interest for robotic and prosthetic applications in the pursuit of a trait that resembles the human skin's ability to repair itself after injury.^90^ In recent years, self‐healing materials were successfully fabricated by mixing healing agents or dynamic reversible bonds, e.g., a metallosupramolecular polymer and a repeatable/healable thermoplastic elastomer material.^91^ However, to achieve high electrical conductivity and repeatable pressure sensitivity of the self‐healing material remains the greatest challenge in meeting the critical demands of these e‐skin applications. Bao and co‐workers pioneered the use of nickel nanostructured particles filled in a hydrogen‐bonded organic supramolecular polymer matrix to develop a flexible and pressure‐sensitive device that possesses self‐healing properties for mechanical and electrical characteristics (**Figure** **14**).[[qv: 11a]] The variation range of the composite resistance increases with decreasing Ni concentration due to the larger changeable space between particles when pressure is applied to the device. The restoration degree of these properties depends on the healing time and ambient temperature. The conductivity could be restored with nearly 90% efficiency after 15 s, thereby allowing promising applications of e‐skin.

**Figure 15:**
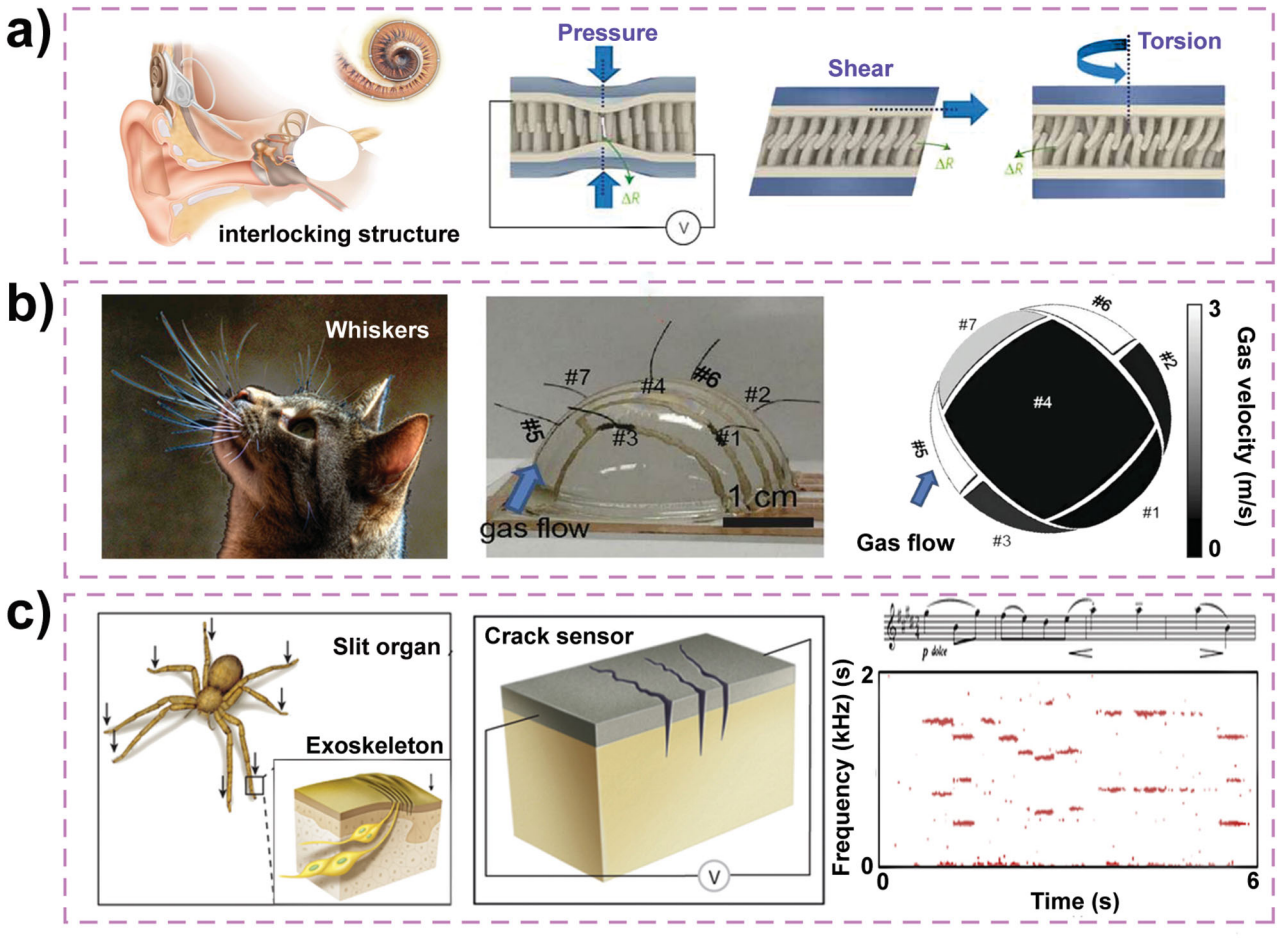
Bio‐inspired design for multi‐force sensing. a) Hair‐to‐hair interlock inspired highly sensitive strain sensor using reversible interlocking of nanofibers. Reproduced with permission.^10^ Copyright 2012, Macmillan Publishers Ltd. b) Whisker inspired wind sensing e‐whiskers. Reproduced with permission.^92^ Copyright 2012, National Academy of Sciences. c) Spider‐sensory‐system‐inspired crack‐based strain and vibration sensor. Reproduced with permission.^19^ Copyright 2014, Macmillan Publishers Ltd.

The development of a multi‐type force‐sensitive sensor that meets the requirements of high sensitivity and flexibility remains a challenge. Inspired by the interlocking biological structures in nature, such as cochlear hair cells, Pang and co‐workers created an ultra‐sensitive multifunctional sensor with two interlocked layers of high‐aspect‐ratio Pt‐coated nanofibers bonded to a flexible substrate (**Figure** **15**a).^10^ The variation of electrical resistance caused by the change in the conducting path between the two arrays was easily measured under different mechanical stimuli, such as pressure, shear, and torsion, and endowed the intelligent e‐skin with a wide range of applications in dynamic signal monitoring in the ultra‐low‐pressure regime. Javey and co‐workers have recently demonstrated the use of highly sensitive and highly bendable electronic whiskers for sensing the 3D distribution of an air flow (Figure 15b).^92^ Two composite electrodes of each whisker bonded to the top and bottom surface of an elastic high‐aspect‐ratio fiber were composed of different concentrations of Ag nanoparticles distributed in a carbon nanotube paste. These electrodes exhibited different changes in resistance to detect varying curvature degrees in different directions. In addition, by mimicking the slit organ configuration of spider legs, Choi and co‐workers created a pressure‐ and vibration‐sensitive sensor based on the nanoscale crack junctions formed in the stretched surface of a metal strip (Figure 15c).^19^ The electrical conductance of the sensor changes with only a small variation in the gaps because of the change in contact resistance of these cracks, which allows the device to detect sound waves for pressure mapping and voice recognition.

**Figure 16:**
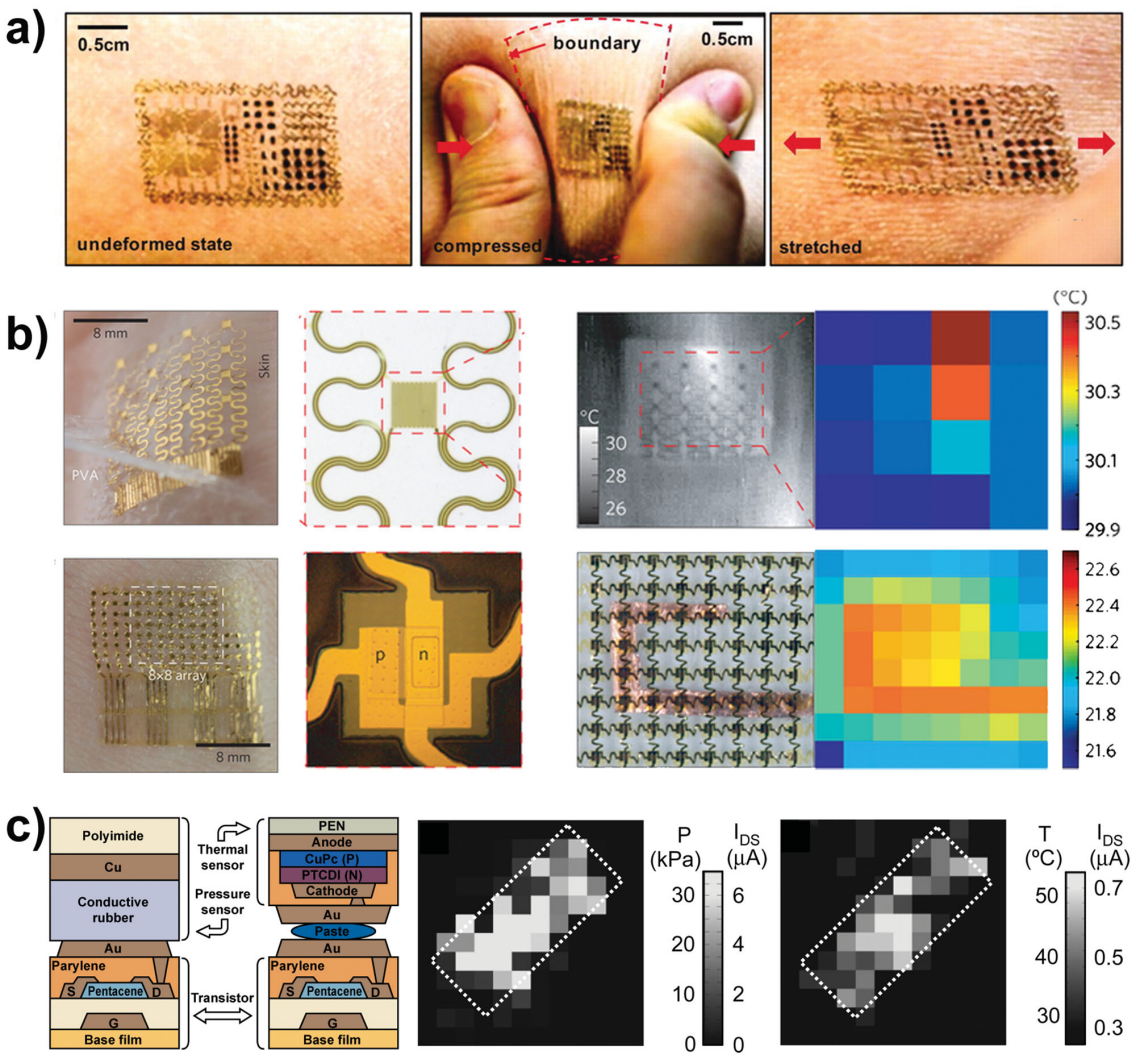
Flexible temperature‐ and pressure‐sensitive sensors. a) Multifunctional epidermal electronic system attached on skin: undeformed (left), crinkled (middle), and stretched (right) states. Reproduced with permission.[[qv: 9a]] Copyright 2011, American Association for the Advancement of Science. b) Ultrathin conformal temperature sensor arrays based on TCR materials and PIN diodes. Reproduced with permission.^95^ Copyright 2013, Macmillan Publishers Ltd. c) Flexible pressure and temperature sensor array based on organic transistors. Reproduced with permission.^99^ Copyright 2005, National Academy of Sciences.

**Figure 17:**
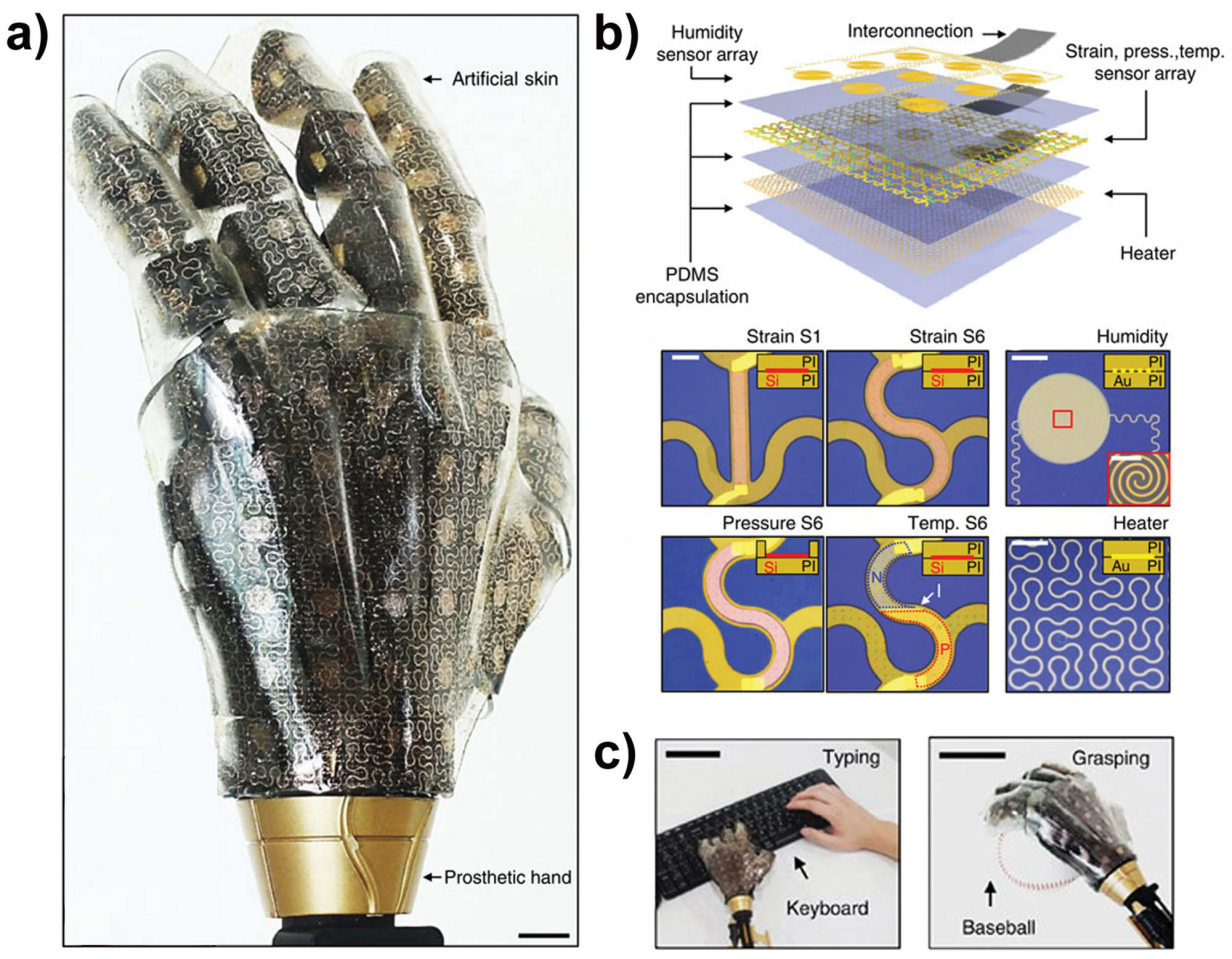
Multi‐functionalized prosthesis with stretchable e‐skin that can sense pressure, temperature, and humidity and can serve as electro‐resistive heaters and stretchable multi‐electrode arrays. a) Photograph of the prosthesis. b) Layered structure of the e‐skin. c) Practical applications of the prosthesis. Reproduced with permission.[[qv: 9b]] Copyright 2014, Macmillan Publishers Ltd.

Temperature‐sensing ability is another important capacity of human skin, which helps to maintain the thermal balance between the body and ambient surroundings and represents a significant breakthrough for the use of temperature‐responsive e‐skin to broaden the applications in human–machine interactions.^93^ The concept of epidermal electronics was first proposed by Rogers and co‐workers, who claimed that multifunctional devices can robustly attach to the uneven surface of the skin via van der Waals forces and can exhibit excellent mechanical and electrical properties for all active components, including sensors for temperature and strain, micro‐scale circuit elements (resistors, capacitors, diodes, transistors, and oscillators) and other auxiliary functional electronic components (light‐emitting diodes, wireless power coils, and radiofrequency generators), as shown in **Figure** **16**a.[[qv: 9a]],^94^ The greatest challenge is to ensure that no detachment phenomena occur after durable deformation cycles. Therefore, lower effective moduli and smaller film thicknesses as well as the design of narrow filamentary serpentine interconnectors have been commonly adopted to reduce the driving force for interface separation. Temperature sensor arrays based on temperature coefficient of resistance (TCR) materials or silicon PIN diodes were demonstrated to continuously and precisely measure the spatial distribution of skin temperature (Figure 16b).^95^ These epidermal electronics were highly sensitive to variations in temperature and thermal conductivity, enabling the evaluation of various human physiological characteristics, such as skin hydration, tissue thermal conductivity, the state of blood flow, and the wound healing process.[[qv: 9a]],^95^, ^96^ By exploiting the use of a PDMS micro‐hair structure, Bao and co‐workers also developed a flexible sensor that can conformably contact irregular skin and provide a variable capacitive response for health monitoring of the temperature and electrocardiogram data via wireless transmission technology.^97^ In addition, a series of multifunctional transistor arrays with pressure and temperature sensitivity has been implemented by several groups.^98^ Someya and co‐workers successfully constructed pressure and thermal active matrixes based on organic transistors to simultaneously read out the distributions of pressure and temperature (Figure 16c).^99^ A PSR layer and organic diodes were laminated together with the transistor film to achieve these functions. Moreover, via direct integration of piezopyroelectric and piezothermoresistive materials into the transistor as a gate dielectric and organic semiconductor channel, Park and co‐workers also fabricated a real‐time multi‐stimuli‐responsive sensor array that could distinguish between the temperature and pressure signals by analyzing the change in the amplitude and offset values of the drain current with AC gate biasing.^100^ Recently, large‐area devices with additional modular components based on a unique sensing mechanism to perceive the complex external environment were subsequently developed to attempt to realistically simulate every outstanding feature of human skin.^101^ Kim and co‐workers implemented an intelligent prosthetic e‐skin that can simultaneously sense temperature, humidity, and multiple forms of strain and is equipped with a heater to adjust the body temperature (**Figure** **17**).[[qv: 9b]] The scientific and precise design of these different sensing element arrays enabled the device to possess fine mechanical reliability and high spatial‐temporal sensitivity to the variable ambient surroundings, indicating that these devices have been well received in a wide range of applications in artificial e‐skin, human–machine interfacing and advanced robotics.

**Figure 18:**
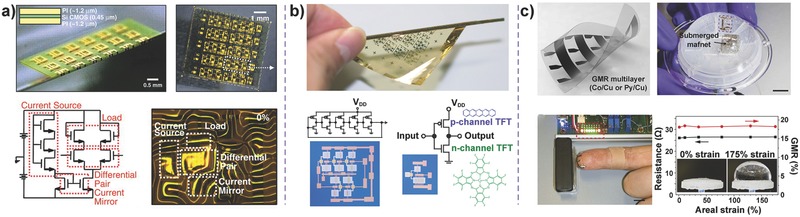
Highly integrated e‐skin systems. a) Stretchable and foldable silicon integrated circuits for e‐skin. Reproduced with permission.^104^ Copyright 2008, American Association for the Advancement of Science. b) Functional organic TFTs and circuits on a flexible ultrathin polymide substrate. Reproduced with permission.^105^ Copyright 2010, Macmillan Publishers Ltd. c) Ultra‐lightweight electronics on ultrathin flexible polymer foil that can sense magnetic fields based on giant magnetoresistance effect. Reproduced with permission.^106^ Copyright 2015, Macmillan Publishers Ltd.

In addition to the functions mentioned above that attempt to mimic human skin, such as strain, pressure, temperature, and humidity sensing, additional features of intelligent e‐skin should be exploited in the future beyond the basic functions and physiognomy of human skin, which are the advantages of e‐skin.^102^ Hence, the high level of integration of the e‐skin system is an urgent topic of investigation, indicating that flexible electronic‐integrated circuits and other chemical or physical sensors should keep pace with the development of e‐skin to meet the demands of applications in robotics, human–machine interactions, health monitoring, and medical implant services.^103^ Rogers and co‐workes implemented complex stretchable, foldable, and high‐quality integrated circuits, including the metal‐oxide‐semiconductor field‐effect transistor (MOSFET), based on single‐crystalline silicon, complementary metal‐oxide semiconductor (CMOS) logic gates, and differential amplifiers (**Figure** **18**a).^104^ These electronics based on inorganic semiconductors and elastomeric substrates simultaneously possess outstanding electrical and mechanical properties. Someya and co‐workers have also demonstrated flexible OFETs and integrated circuits on an ultrathin polymer substrate, including complementary inverters and ring oscillators of which the bending radius is notably as small as 100 μm without a decline in electrical characteristics (Figure 18b).^105^ Considering the potential application of lightweight devices, certain types of imperceptible electronics were further investigated. An ultrathin and lightweight magnetic field sensor based on the giant magnetoresistance effect was successfully constructed to detect static or dynamic magnetic fields (Figure 18c).^106^ Other imperceptible electronics, such as implantable electronics and adhesive sensors, were also developed to detect various biological signals from body tissue.^107^

### 4.3 Wireless Technology for E‐Skin

Wireless communication is commonly used in our daily lives. This communications approach can eliminate the restrictions of disorganized wires and cables and enables the transfer of information between unconnected objects. To exploit wireless technology, certain research groups have pursued applications of e‐skin sensors for wireless sensor arrays, wireless energy transfer, and wireless data communication.^108^

**Figure 19:**
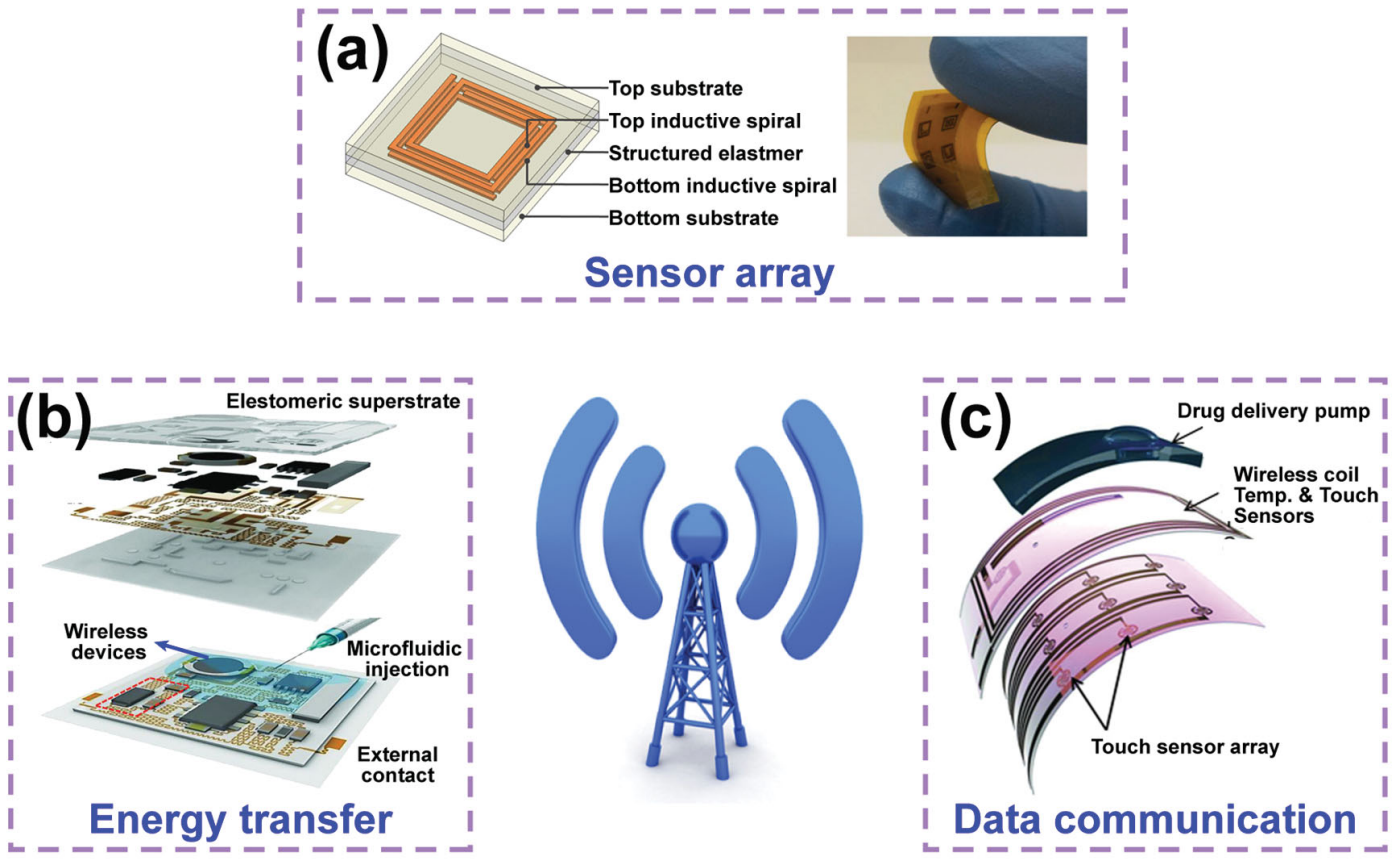
Applications of wireless technology on e‐skin. a) Flexible wireless pressure sensor array. Reproduced with permission.[[qv: 16c]] Copyright 2014, Macmillan Publishers Ltd. b) Wireless energy‐transfer module for e‐skin. Reproduced with permission.^46^ Copyright 2014, American Association for the Advancement of Science. c) Touch and temperature sensor array integrated with wireless data transmission module. Reproduced with permission.^111^

Bao and co‐workers demonstrated a flexible wireless sensor array that can monitor and map the pressure variation in real time (**Figure** **19**a).[[qv: 16c]] The resonant circuit of the device consists of a pressure‐dependent capacitive element and an inductive antenna in which the resonant frequency decreases with applied pressure due to the increase in effective coupling capacitance. This wireless detection strategy with a lower frequency than other traditional schemes enables wider applications in human–machine interactions, intracranial pressure monitoring, and biomedical research. Rogers and co‐workers equipped the wireless energy‐transfer module with epidermal electronics that included tiny commercial chips, integrated circuits, and various sensors for accurate monitoring and precise measuring in clinical settings using a wireless mode (Figure 19b).^46^ Recently, an implantable and biodegradable electronics‐based device on a Mg wireless heater was developed that can be fully degraded in water to realize remote‐control therapy in vivo, which could offer the possibility of heat disinfection near an operative wound and triggering of drug release.^109^ Based on wireless data communication, Bao and co‐workers created a flexible and highly sensitive temperature sensor to remotely monitor the temperature of the human body. The device is composed of a passive radiofrequency identification antenna and a resistance‐responsive composite film consisting of Ni particles filled within a polyethylene/polyethylene oxide binary matrix polymer.^110^ A smart bandage based on MEMS with various sensors and a wireless signal transmission component was explored to complete the drug delivery function of the wearable and flexible device (Figure 19c).^111^ Other flexible and wearable devices integrated with transistors and electromyogram and electrocardiogram sensors were also reported to monitor human body health and to provide the foundation for widespread application of large‐scale integrated wearable devices in human–machine interactions via wireless communication.[[qv: 9a]],^108^

## 5 Conclusions and Outlook

In this review, we highlight the primary approaches used to construct more flexible and stretchable sensors and the efforts toward the delivery of high‐performance e‐skin. Recent noteworthy achievements in the use of functional materials and the optimization of device design are convincing solutions to mimic the unique features of human skin. The pressure sensitivity of sensors is dramatically enhanced with the use of transistors with microstructured gate dielectrics, which enables the active matrix to reduce signal crosstalk between pixels and promotes rapid addressing and low‐power consumption. In addition, the use of oriented piezoelectric NWs and NBs with high intrinsic piezoelectricity and good mechanical stability accelerates the development of high‐resolution sensing arrays that extend beyond the capabilities of human sensing. Moreover, the fabrication of multifunctional e‐skins is an essential research goal to satisfy industry requirements for various applications. Highly integrated electronics for the detection of multiple stimuli are the subject of many investigations. With the aid of newly emerging technologies, e.g., wireless technology and the preparation of ultrathin sensors, these efforts have received substantial attention in the field of health monitoring and medical implant services.

However, certain challenges remain for practical applications. For example, new materials and a novel transduction mechanism should be further investigated to realize a tunable pressure measurement range. Although ultra‐sensitive sensors have been reported in recent studies, the pressure measurement range should be further expanded to satisfy the demands of various applications. Moreover, the production of devices with low‐power consumption or self‐powering ability remains a topic worthy of in‐depth study because the energy crisis is currently one of the largest challenges in our society. Additionally, different types of artificial smart e‐skins are urgently required for emerging fields in healthcare, including health monitoring, prosthesis techniques, and clinical medicine. Similar to real human skin that can adjust and provide feedback in real time according to the different types of external stimuli via the peripheral nervous system, future e‐skins will also intelligently respond to variations in the external environment based on novel information transmission technology, which has a bright future for the development of e‐skins.
